# Tackling Imbalanced Data in Chronic Obstructive Pulmonary Disease Diagnosis: An Ensemble Learning Approach with Synthetic Data Generation

**DOI:** 10.3390/bioengineering13010105

**Published:** 2026-01-15

**Authors:** Yi-Hsin Ko, Chuan-Sheng Hung, Chun-Hung Richard Lin, Da-Wei Wu, Chung-Hsuan Huang, Chang-Ting Lin, Jui-Hsiu Tsai

**Affiliations:** 1 Department of Computer Science and Engineering, National Sun Yat-sen University, Kaohsiung 804, Taiwan; zxc78945606nsysu@gmail.com (Y.-H.K.); lin@cse.nsysu.edu.tw (C.-H.R.L.); m113040003@nsysu.edu.tw (C.-H.H.); linctin05@cgmh.org.tw (C.-T.L.); 2 Artificial Intelligence Research and Promotion Center, National Sun Yat-sen University, Kaohsiung 804, Taiwan; 3 Research Center for Precision Environmental Medicine, Kaohsiung Medical University, Kaohsiung 807, Taiwan; u8900030@gmail.com; 4 Faculty of Medicine, College of Medicine, Kaohsiung Medical University, Kaohsiung 807, Taiwan; 5 Department of Internal Medicine, Kaohsiung Municipal Siaogang Hospital, Kaohsiung Medical University Hospital, Kaohsiung Medical University, Kaohsiung 812, Taiwan; 6 Division of Pulmonary and Critical Care Medicine, Department of Internal Medicine, Kaohsiung Medical University Hospital, Kaohsiung Medical University, Kaohsiung 807, Taiwan; 7 Teaching and Research Center of Kaohsiung Municipal Siaogang Hospital, Kaohsiung 812, Taiwan; 8 Division of Hematology and Oncology, Kaohsiung Chang Gung Memorial Hospital, Kaohsiung 833, Taiwan; 9 School of Medicine, Tzu Chi University, Hualien 970, Taiwan; 10 Department of Psychiatry, Dalin Tzu Chi Hospital, Buddhist Tzu Chi Medical Foundation, Chia-Yi 622, Taiwan

**Keywords:** data imbalance, COPD, machine learning, tabular generative adversarial networks, kernel density estimation, ensemble learning, stacking

## Abstract

Chronic obstructive pulmonary disease (COPD) is a major health burden worldwide and in Taiwan, ranking as the third leading cause of death globally, and its prevalence in Taiwan continues to rise. Readmission within 14 days is a key indicator of disease instability and care efficiency, driven jointly by patient-level physiological vulnerability (such as reduced lung function and multiple comorbidities) and healthcare system-level deficiencies in transitional care. To mitigate the growing burden and improve quality of care, it is urgently necessary to develop an AI-based prediction model for 14-day readmission. Such a model could enable early identification of high-risk patients and trigger multidisciplinary interventions, such as pulmonary rehabilitation and remote monitoring, to effectively reduce avoidable early readmissions. However, medical data are commonly characterized by severe class imbalance, which limits the ability of conventional machine learning methods to identify minority-class cases. In this study, we used real-world clinical data from multiple hospitals in Kaohsiung City to construct a prediction framework that integrates data generation and ensemble learning to forecast readmission risk among patients with chronic obstructive pulmonary disease (COPD). CTGAN and kernel density estimation (KDE) were employed to augment the minority class, and the impact of these two generation approaches on model performance was compared across different augmentation ratios. We adopted a stacking architecture composed of six base models as the core framework and conducted systematic comparisons against the baseline models XGBoost, AdaBoost, Random Forest, and LightGBM across multiple recall thresholds, different feature configurations, and alternative data generation strategies. Overall, the results show that, under high-recall targets, KDE combined with stacking achieves the most stable and superior overall performance relative to the baseline models. We further performed ablation experiments by sequentially removing each base model to evaluate and analyze its contribution. The results indicate that removing KNN yields the greatest negative impact on the stacking classifier, particularly under high-recall settings where the declines in precision and F1-score are most pronounced, suggesting that KNN is most sensitive to the distributional changes introduced by KDE-generated data. This configuration simultaneously improves precision, F1-score, and specificity, and is therefore adopted as the final recommended model setting in this study.

## 1. Introduction

The global and Taiwan burden of chronic obstructive pulmonary disease (COPD) is recognized as a major public health issue, ranking as the third leading cause of death worldwide, responsible for more than 3.2 million deaths annually and affecting over 200 million people globally [1]. This persistently heavy global disease burden reflects long-term exposure to major risk factors such as tobacco smoking, indoor biomass fuel use, and ambient air pollution, a situation that is particularly pronounced in low- and middle-income countries with limited healthcare resources [2]. According to the Global Burden of Disease Study, COPD accounted for approximately 74 million disability-adjusted life years (DALYs) in 2019, representing one of the most important contributors to chronic disease-related morbidity and mortality worldwide [3].

In Taiwan, COPD is an important chronic respiratory disease, and its prevalence has continued to increase over the past two decades. A nationwide epidemiological survey reported that among adults aged 40 years and older, the prevalence of COPD rose from 2.5% in 1996 to 6.1% in 2015, with a substantial proportion of cases remaining undiagnosed or inadequately treated [4]. These findings indicate that smoking habits and ambient air pollution play a critical role in disease progression and prognosis. Overall, evidence from both global and Taiwanese data emphasizes that COPD remains a central target for early detection, effective control of risk factors, and long-term disease management strategies to mitigate its growing health and socioeconomic burden.

From the perspectives of the patient level and the healthcare system, short-term readmission of patients with COPD within 14 days after discharge is a key indicator reflecting disease instability and inefficiencies in the healthcare system. Evidence from real-world studies and systematic literature reviews indicates that such early readmissions are driven by multiple factors, encompassing both patient vulnerability and gaps in transitional care within the healthcare system.

At the patient level, multiple studies have identified several determinants associated with short-term readmission among individuals with COPD, including lower lung function after discharge, such as reduced ${\mathrm{FEV}}_{1}$), long-term corticosteroid use, multiple comorbidities (e.g., cardiovascular or renal diseases), and a history of prior hospitalizations [5]. These factors suggest that patients discharged while their physiological condition remains unstable or incompletely recovered are at higher risk of relapse within two weeks after discharge. In addition, frequent acute exacerbations and persistent systemic inflammation may lead to poor symptom control and a vicious cycle of early recurrence [6].

From the healthcare system perspective, frequent early readmissions reflect insufficient transitional care and impose substantial economic and operational burdens. Di Chiara et al. [7] reported that recurrent hospitalizations for COPD account for a considerable proportion of healthcare expenditures and bed occupancy, while also exposing patients to a higher risk of nosocomial infections and complications related to polypharmacy. Several systematic analyses have estimated that 30-day readmission rates are approximately 15–25% and can reach 40% within 90 days, suggesting that 14-day readmissions likely represent a subset of recurrent events with high preventability [6]. In hospitals where discharge planning is inadequate or integration between outpatient and community care is insufficient, short-term relapse rates are often higher, highlighting the necessity of establishing timely and continuous care pathways, remote monitoring, and integrated community-based care to reduce avoidable readmissions.

To reduce readmissions within 14 days, it is essential to identify patients whose physiological status remains unstable before discharge by integrating biomarkers, symptom monitoring, and assessments of medication adherence. Subsequently, multidisciplinary team interventions, such as pulmonary rehabilitation, home oxygen monitoring, and digital follow-up and tracking, should be initiated to maintain clinical stability after discharge. Well-designed preventive strategies can not only improve survival and quality of life but also enhance the efficiency of healthcare resource utilization and the sustainability of the healthcare system [7].

Hospital readmission following an acute exacerbation of COPD remains an important indicator of disease instability and quality of care. Despite continuous advances in therapeutic options, readmission rates remain high due to multiple contributing factors. Systematic reviews have indicated that comorbidity burden, prior hospitalization history, and longer length of stay are major determinants of all-cause readmission [6]. Similarly, short-term readmission and 1-year mortality risk are closely associated with advanced age, impaired lung function, and hypoxemia, underscoring the early post-discharge period as a highly vulnerable and critical window [5].

Recent evidence indicates that readmission events are highly concentrated within the first 14 days after discharge, with the majority of preventable recurrences accumulating within this “high-risk window” [7]. Conventional prediction models that focus on 30-day or 90-day readmission may overlook this extremely critical early period. By establishing a prediction model specifically targeting 14-day readmission risk, healthcare teams can initiate interventions earlier, such as remote monitoring, adherence support, and timely follow-up visits.

In recent years, the importance of artificial intelligence (AI) has continued to rise. Among AI technologies, machine learning, as a core component, has been widely applied across diverse domains [8]. However, in practical training settings, constraints such as limited sample size and class imbalance often cause learning to be dominated by the majority class, leading to underfitting characterized by high bias and low recall for the minority class, which in turn undermines model generalizability and robustness [9]. Class imbalance is prevalent in many fields [10] and the problem is particularly pronounced in healthcare. Owing to privacy protection and institutional review regulations, access to and sharing of clinical data are highly restricted, further exacerbating data scarcity and class imbalance. Synthetic data have been considered an effective means to address challenges related to data access, privacy, and confidentiality; consequently, their potential and value have received increasing attention [11]. Strategies for mitigating class imbalance can be broadly categorized into three groups: data-level techniques, algorithm-level techniques, and hybrid approaches [10]. This study adopts a hybrid approach because the 14-day readmission prediction task exhibits substantial class imbalance, and relying on a single level of intervention (data-level only or algorithm-level only) is often insufficient to simultaneously ensure minority-class discriminability and model robustness. Therefore, we integrate both data-level and algorithm-level strategies into a unified hybrid pipeline tailored to the task requirements.

At the data level, we use KDE/CTGAN during training to augment the minority class, improving minority coverage in the feature space and reducing the risk of minority-class underfitting by downstream classifiers. Compared with conventional oversampling (e.g., random duplication) or purely interpolation-based methods (e.g., SMOTE), generative augmentation can provide more diverse samples and thereby mitigate overfitting caused by duplicated instances or decision-boundary distortion induced by linear interpolation.

At the algorithm level, we employ stacking ensemble learning to integrate complementary signals from multiple base learners. Compared with a single classifier (e.g., a single Random Forest or a single SVM), stacking can more stably preserve minority-class recognition capability under class imbalance, enabling more reliable assessment of COPD patients’ readmission risk.

Moreover, machine learning models have been shown to offer advantages in predictive accuracy [12]. In COPD care, intelligent clinical decision support systems have demonstrated the potential to reduce unplanned readmissions through individualized risk estimation [13]. Although prediction models for survival outcomes following acute exacerbations have been developed [14], AI models specifically targeting short-term readmission remain relatively scarce. For example, Nhu et al. [15] successfully applied survival analysis-based machine learning models to predict 14-day readmission risk in patients with pneumonia, suggesting the feasibility of applying such approaches to COPD.

Therefore, developing an AI prediction model specifically targeting the 14-day readmission risk among patients with COPD is both necessary and practically feasible. Such a model can strengthen precision medicine by enabling healthcare teams to intervene at an earlier stage, reduce avoidable hospitalizations, improve patient outcomes, and enhance the efficiency of the healthcare system.

Accordingly, this study integrates real-world clinical data from the Kaohsiung Medical University Hospital system, Kaohsiung Municipal Siaogang Hospital, and Kaohsiung Municipal Ta-Tung Hospital. To address the pronounced class imbalance, we adopt kernel density estimation (KDE) and conditional tabular generative adversarial networks (CTGANs) as data generation strategies and systematically evaluate their applicability and predictive performance on this dataset. Specifically, we first construct predictions using multiple base models and then employ stacking to aggregate their outputs into the final decision. Finally, we conduct ablation experiments by progressively removing each base model to quantify its individual contribution and compare the results with other baseline models to demonstrate the greater generalizability and stability of stacking. The contributions of this work are threefold: (1) under an imbalanced clinical data setting, we compare the impact of the generative quality of KDE and CTGAN on predictive performance; (2) we elucidate the relative advantages of different base models when trained with synthetic data; and (3) through stacking and an ablation design, we identify the optimal generation strategy and quantify model contributions, thereby confirming the stability of stacking for predicting the risk of 14-day readmission among patients with COPD.

## 2. Related Works

In recent years, artificial intelligence has developed rapidly, and machine learning and deep learning have been widely applied to risk prediction and admission decision support in chronic obstructive pulmonary disease (COPD). A review of existing studies shows that COPD prediction commonly faces the challenge of class imbalance and often involves comparing multiple machine learning models or applying deep learning models for hospitalization-related prediction, while simultaneously examining the key variables identified by these models. Accordingly, the following sections review and discuss the relevant literature on the application of machine learning and deep learning to COPD prediction.

Mohamed et al. [16] proposed a data-analytic workflow using single-center COPD hospitalization records to construct 30- and 90-day readmission prediction models. After data cleaning and integration, 195 records with 32 variables were obtained. The authors developed models using artificial neural networks (ANN, including MLP and RBF), decision trees (CHAID, C5.0, C&RT), and multi-kernel SVM, and compared the predictability and influencing factors for readmission within 1 month and within 3 months. Model performance was evaluated using the area under the ROC curve (AUC) and accuracy (ACC), with cross-validation applied to ensure generalizability. The study showed that, under the shorter prediction window (readmission within 1 month), the models exhibited better discriminative ability, with CHAID/exhaustive CHAID achieving an AUC of up to 0.77 and C5 reaching an ACC of 89.9%, indicating that 30-day readmission risk can be effectively and relatively accurately predicted. In contrast, prediction over a 3-month (approximately 90-day) window was clearly more challenging: the best ANN-RBF model achieved an AUC of only 0.64, and the highest ACC of CHAID/exhaustive CHAID was 67.7%, indicating that the accuracy and stability of medium-term readmission prediction are more limited and that larger case numbers and additional risk factors are needed to further strengthen the models.

Lopez et al. [17] developed a readmission prediction model using EHR data from index hospitalizations between 2012 and 2017. From 5794 inpatients, the authors constructed two major datasets comprising 777 patients with asthma and 1905 patients with COPD, and evaluated four machine learning models (Naive Bayes, SVM, Random Forest, Gradient-Boosted Trees) and one deep learning model (Multilayer Perceptron, MLP). Model performance was assessed using AUC, precision–recall (PR) metrics, and SHAP (Shapley Additive explanation, SHAP) to analyze feature contributions. In the combined dataset, all five models achieved an AUC greater than 0.83; under class-imbalanced conditions, Naive Bayes showed poorer average precision (AP). Compared with the four traditional machine learning (ML) models, MLP achieved the best balance between sensitivity and specificity and was more effective in identifying readmitted cases. In terms of feature importance, SHAP analysis indicated that the top 10 key features included hematologic indices (WBC, ANC, ALC, MPV), in-hospital procedures and medications (ICS/LABA, antibiotics, albuterol, ipratropium, pneumococcal vaccination), and CHF. Among these, elevated white blood cell counts and higher mean platelet volume contributed predominantly negative effects, whereas higher neutrophil and lymphocyte counts were associated with positive contributions. Overall, in the combined dataset, MLP demonstrated a better trade-off between sensitivity and specificity, and the SHAP analysis recovered several known characteristics of “frequent exacerbators.” These findings support the feasibility of integrating deep learning with EHR data to prioritize care for high-risk patients and highlight the potential for integration with clinical decision support systems.

## 3. Architecture

This study aims to address the critical machine learning challenge of imbalanced classification, using clinical prediction of chronic obstructive pulmonary disease (COPD) as the application case. By combining data generation methods with ensemble learning strategies, we seek to train a classification model that exhibits high stability and strong predictive performance for the minority class. To this end, we first rigorously define the problem scope, data structure, characteristics of the imbalanced data, and the final optimization objective.

Let the complete dataset be denoted by *D*, where each sample d consists of an m dimensional feature vector *X* and a binary label *y*. We partition *D* into three mutually exclusive subsets: the training set $D_{\mathrm{train}}$, the validation set $D_{\mathrm{valid}}$, and the final test set $D_{\mathrm{test}}$:

(1) $$\begin{array}{c} D=D_{\mathrm{train}}\;\cup \;D_{\mathrm{valid}}\;\cup \;D_{\mathrm{test}}\;\mathrm{and}\;D_{\mathrm{train}}\;\cap \;D_{\mathrm{valid}}\;\cap \;D_{\mathrm{test}}=\emptyset \end{array}$$

The structure of each subset is defined as follows:

$$\begin{array}{ccc} D_{\mathrm{train}} & ={\left\{\left(X_{i},y_{i}\right)\right\}}_{i=1}^{I}, & \;\;\;X_{i}\;\in \;R^{m},y_{i}\;\in \;\left\{0,1\right\},\left|D_{\mathrm{train}}\right|=I\\ D_{\mathrm{valid}} & ={\left\{\left(X_{j},y_{j}\right)\right\}}_{j=1}^{J}, & \;\;\;X_{j}\;\in \;R^{m},y_{j}\;\in \;\left\{0,1\right\},\left|D_{\mathrm{valid}}\right|=J\\ D_{\mathrm{test}} & ={\left\{\left(X_{k},y_{k}\right)\right\}}_{k=1}^{K}, & \;\;\;X_{k}\;\in \;R^{m},y_{k}\;\in \;\left\{0,1\right\},\left|D_{\mathrm{test}}\right|=K \end{array}$$

Here, *I*, *J*, and *K* denote the total numbers of samples in the training, validation, and test sets, respectively, and mmm is the dimensionality of the feature vector. The label *y* ∈ {0,1} defines the two classes of the binary classification problem. For the binary classification setting, the complete dataset *D* can be further partitioned by label into the majority class $D_{\mathrm{major}}$ (e.g., *y* = 0), and the minority class $D_{\mathrm{minor}}$ (e.g., *y* = 1).

(2) $$\begin{array}{cccc} D & =D_{\mathrm{major}}\;\cup \;D_{\mathrm{minor}}, & \;\;\;D_{\mathrm{major}}\;\cap \;D_{\mathrm{minor}} & =\emptyset \end{array}$$

Due to the presence of a highly skewed class imbalance in the dataset, such that the sample sizes satisfy $|D_{\mathrm{major}}|$ ≫ $|D_{\mathrm{minor}}|$, conventional classification models tend to be biased toward predicting the majority class during training, which in turn leads to a substantial degradation in predictive performance and generalization ability for the critical minority class.

The overall algorithm *A* designed in this study combines data generation methods (such as KDE or CTGAN) with an ensemble learning model $M=\{m_{1},\ldots ,m_{n}\}$. In practice, we train candidate models on $D_{\mathrm{train}}$ and use the validation set $D_{\mathrm{valid}}$ to compare the predictive performance of different data generation schemes, feature combinations, and model architectures, in order to select the configuration that performs best on the minority class (14-day readmission).

Let ω denote the set of configurations to be optimized, encompassing the data generation strategy, feature subset, ensemble architecture, and classification threshold. Our model selection objective is to identify the optimal configuration $\omega^{*}$, that maximizes the overall classification performance metric, Performance, on the validation set $D_{\mathrm{valid}}$:

(3) $$\begin{array}{c} \omega^{*}=\arg\max_{\omega }\;\mathrm{Performance}\left({\left\{\left(A_{\omega }\left(X_{j}\right)\right),y_{j}\right\}}_{j=1}^{J}\right) \end{array}$$

Here, Performance(·) is used to evaluate the overall predictive quality under imbalanced classification. In this study, we particularly focus on metrics such as the F1-score, the area under the ROC curve (AUC), and the maximum precision at fixed recall thresholds, in order to ensure that the model maintains stable and reliable discriminative ability for the minority class.

Finally, we apply the selected optimal model $A_{\omega^{*}}$ to the independent test set $D_{\mathrm{test}}$ to evaluate its generalization ability, and take these results as the final evaluation outcome of this study.

## 4. Method

### 4.1. Data Preprocessing and Data Augmentation

**Figure 1:**
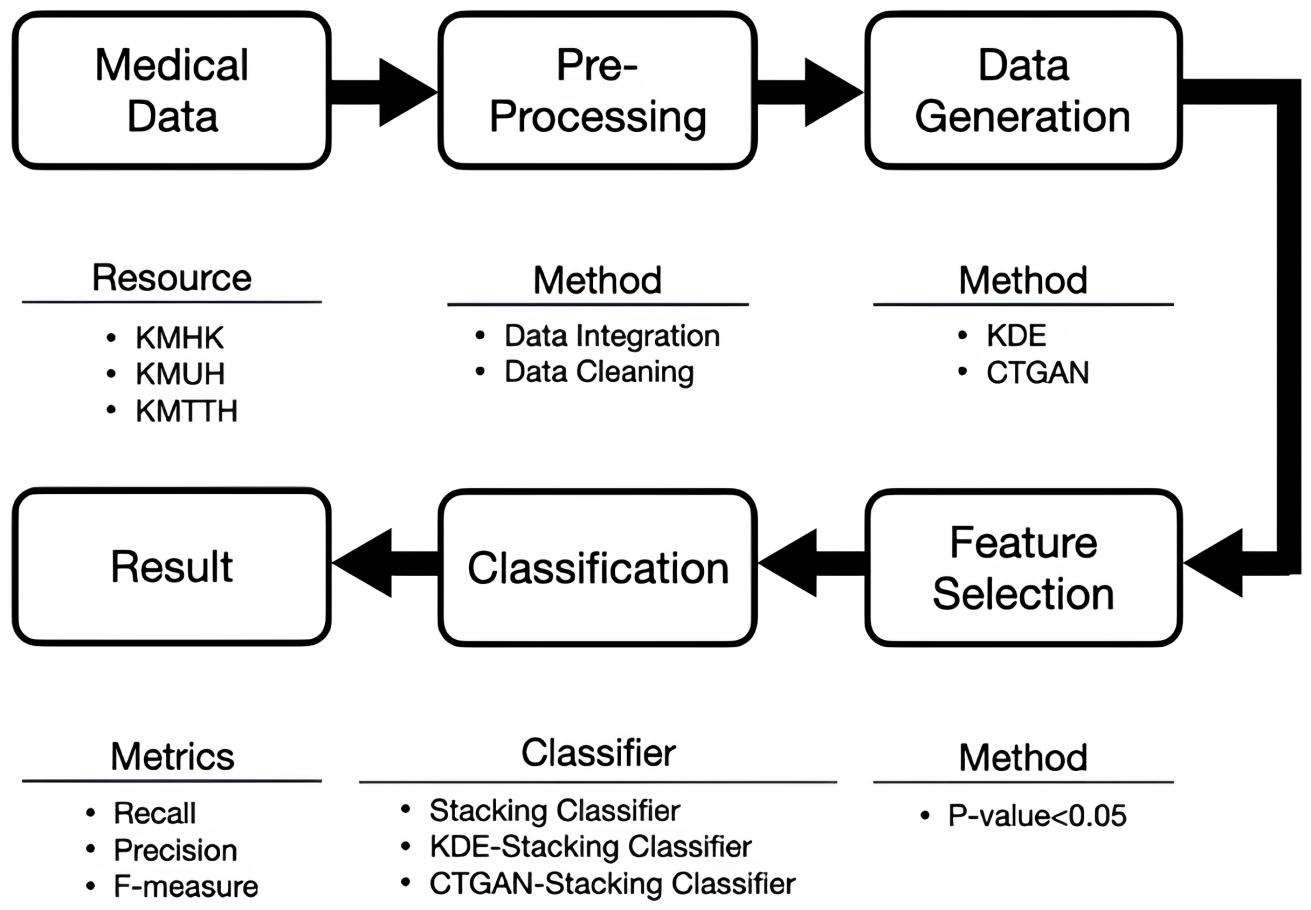
Study framework.

The overall research framework of this study is illustrated in Figure 1. The prediction target is to determine whether a hospitalized COPD case is readmitted within 14 days after discharge due to the same underlying cause. Accordingly, an unplanned readmission within 14 days after discharge is defined as a positive sample, whereas cases with an interval longer than 14 days or without any subsequent admission are defined as negative samples. Thus, we define unplanned readmission within 14 days after discharge as y=1, and an interval exceeding 14 days or no readmission as y=0.

Operationally, we used each discharge date as the index point and reviewed subsequent hospitalization records over the following 14 days. If a readmission meeting the study criteria occurred within this 14-day window, the case was labeled y=1; otherwise, it was labeled y=0. Labels were assigned based on date fields. In addition, to prevent data leakage arising from multiple admissions of the same patient, we performed dataset splitting at the patient level using patient ID as the grouping unit, ensuring that all hospitalization records for a given patient appeared in only one data subset.

A total of 94 predictors were included, covering sex, age, comorbidities, medications, vaccination, laboratory test results, and air quality indicators. The data were derived from retrospective records (2012–2020) from three hospitals within the Kaohsiung Medical University healthcare system in Taiwan: Kaohsiung Medical University Hospital, Kaohsiung Municipal Siaogang Hospital, and Kaohsiung Municipal Ta-Tung Hospital. We integrated multiple sources, including inpatient claims files, in-hospital mortality registration files, patient demographic data, self-paid outpatient procedures and medication files, laboratory report files, and historical air quality monitoring data from the Ministry of Environment. The study population comprised hospitalized cases aged 18–100 years.

We first extracted COPD hospitalization records from the inpatient claims files and in-hospital mortality registration files. Because the study period spans both ICD-9-CM and ICD-10-CM coding eras, we applied two COPD-related code sets (ICD-9-CM and ICD-10-CM) and performed harmonization across coding generations. Specifically, diagnosis codes from different eras were mapped to the same COPD diagnostic category according to predefined correspondence rules to ensure consistent case identification across years. We then compiled care-history indicators, including the number of previous admissions, length of stay, and the interval since the previous admission, and assigned positive/negative labels for 14-day readmission based on date fields. Considering the influence of environmental exposure on COPD exacerbation, we selected the nearest air-quality monitoring station according to the patient’s residential address (and, where applicable, also considering sex and age to align with community distribution and healthcare-seeking geography). We incorporated the 30-day moving average of pollutant concentrations prior to discharge to reflect individualized exposure levels.

Medication and vaccination data were obtained from the self-paid outpatient procedure and medication files. For medications, we summarized the actual drugs administered during hospitalization and used them to grade COPD severity. For vaccinations, we indicated whether the patient had completed relevant vaccinations prior to the index admission in order to assess their association with preventive interventions. Blood test results were extracted from laboratory report files. To align with the temporal sequence of clinical decision-making and to avoid dependence between samples and overfitting caused by repeated tests during the same hospitalization, we uniformly used a single result—the one closest to the discharge date—as the representative value. Given the large number of laboratory items and the presence of structural missingness, directly discarding records with missing values would substantially reduce the effective sample size and introduce selection bias. On the recommendation of clinical physicians, missing laboratory values were imputed using random values within the clinically normal range to preserve the variability structure of the data and reduce the risk of systematic bias.

To balance data completeness and clinical interpretability, no additional feature engineering was performed beyond necessary cleaning and missing-value handling, so as to avoid over-processing that could dilute the original signal and to reduce preprocessing costs. Because the incidence of readmission within 14 days after discharge was low, the training data were severely imbalanced; to enhance discrimination of the minority class, this study adopted an oversampling strategy, applying two tabular data generation methods—CTGAN [18] and kernel density estimation (KDE) [19]—to augment positive samples in the training set, thereby adjusting the original negative-to-positive ratio from 1:14 to approximately 1:4. No synthesis or resampling was applied to the validation or test sets in order to avoid data leakage and to preserve the true prevalence as an objective evaluation benchmark.

CTGAN excels at generating data with complex structures and dependency relationships, and can simultaneously handle categorical and continuous variables while preserving the distribution of categorical features, thereby capturing nonlinear relationships and complex distributions in the data. It is particularly well suited for high-dimensional and structurally complex datasets. However, the generation process of CTGAN is relatively complex, its outputs are comparatively less interpretable, and it requires higher computational costs and longer training time. To enhance the stability of adversarial training and prevent mode collapse, its loss function adopts the WGAN loss with gradient penalty (WGAN-GP) for model training [18]; please refer to Equation (4) [20]

(4) $$\begin{array}{c} L=E_{G\left(z\right)∼P_{g}}\left[D(G(z))\right]-E_{x∼P_{r}}\left[D(x)\right]+\lambda \;E_{\hat{x}∼P_{\hat{x}}}\left[(∥\nabla_{\hat{x}}D\left(\hat{x}\right){∥-1)}^{2}\right] \end{array}$$

KDE, by contrast, is a nonparametric method that generates new samples by estimating the probability density function of the data. Its implementation is relatively simple and the computation process is intuitive, making it suitable for handling continuous variables. However, its ability to deal with categorical data is limited, and its performance is also constrained when facing nonlinear features. The data generated by KDE are relatively smooth and more appropriate for scenarios with comparatively simple distributional structures, and both its computational cost and training time are substantially lower than those of CTGAN.

(5) $$\begin{array}{c} {\hat{f}}_{h}\left(x\right)=\frac{1}{nh^{d}}\sum_{i=1}^{n}K\left(\frac{x-x_{i}}{h}\right) \end{array}$$

Equation (5) gives the KDE formulation, where *x* denotes the point at which the density is to be evaluated, $x_{i}$ is the *i*-th observation, *d* is the dimensionality of the feature space, K(·) is the kernel function, and the bandwidth h>0 is the smoothing parameter. The resulting KDE can be interpreted as assigning, around each observation $x_{i}$, a local weighting function determined by K(·) and *h* and then taking a weighted average over all observations to obtain a continuous and smooth estimated density function $\hat{f_{h}}\left(x\right)$.

The core challenge of our data characteristics lies in the following: our objective is to augment the minority class of 14-day readmission (reADM = 1), while the minority cohort in this dataset simultaneously exhibits two common yet conflicting requirements. First, most continuous measurements follow clearly non-Gaussian distributions (skewed, heavy-tailed, and even multimodal), and clinically relevant signals associated with short-term deterioration risk often concentrate in the tail or extreme-value regions. Second, the dataset contains a large number of sparse 0/1 features (comorbidities and medications) with strong clinical co-occurrence dependencies, such that they cannot be generated by independently sampling each field and arbitrarily combining them.

Accordingly, we treat KDE and CTGAN as two independent generative strategies and compare them because each directly targets a different difficulty described above. KDE is primarily suitable because it performs neighborhood-based, nonparametric sampling centered on real minority instances; generated points typically fall within the local density region of the minority cohort. This property is advantageous for preserving tail coverage and multimodal shapes in continuous features, thereby reducing the risk that generated values shrink toward the overall mean and dilute extreme minority signals. Meanwhile, KDE’s limitations are consistent with our data characteristics: when feature dimensionality is high and interactions are complex, KDE tends to provide “local fidelity” rather than fully learning higher-order dependencies across features. Therefore, in this study, KDE is evaluated mainly for its ability to enhance minority-neighborhood coverage and preserve tail fidelity. We first represent categorical variables in numeric form and fit a joint KDE over both categorical and continuous variables, followed by sampling from the estimated joint distribution. Because KDE sampling outputs continuous values, categorical variables are discretized using a 0.5-step rule and mapped to the nearest valid category (e.g., for categories 1–4, thresholds are 1.5, 2.5, and 3.5).

In contrast, CTGAN is primarily suitable due to its conditional generation framework and discrete-field handling mechanisms designed for mixed-type tabular data. Under minority-conditioned generation, CTGAN can more directly learn and preserve the structural patterns and clinical co-occurrence relationships of sparse 0/1 features, mitigating co-occurrence dilution or clinically implausible combinations that may arise when discretizing generated outputs. Its limitation is that adversarial training can be sensitive to hyperparameters and minority sample size; when the minority class is extremely small, coverage of rare combinations may remain insufficient. We therefore evaluate its robustness under the same targeted augmentation setting and downstream performance criteria.

We also considered TVAE. Compared with KDE and CTGAN, TVAE, as a reconstruction-driven VAE-based tabular generator, is advantageous in terms of relatively stable training and providing a conservative baseline for global distribution reconstruction. However, because its objective includes KL regularization that encourages a continuous and smooth latent representation, under the native non-conditional setting, the model tends to reflect the average structure of the overall population. When the goal is targeted augmentation for the minority class, this tendency may lead to insufficient coverage of minority tail signals. Moreover, under a large number of sparse 0/1 fields, the decoder typically outputs probabilities that are then discretized; without additional designs or constraints tailored to sparsity and co-occurrence dependencies, the clinical co-occurrence logic of comorbidities/medications may be diluted or yield clinically less plausible combinations.

In summary, CTGAN and KDE address the two key requirements, respectively. CTGAN is more capable of learning nonlinear cross-feature dependencies in mixed-type tabular data and of preserving the structure of sparse features such as comorbidities and medications via conditional generation and discrete-field mechanisms. KDE, by contrast, provides locally density-based smooth augmentation around minority instances, which helps improve minority coverage in the tails and multimodal regions of continuous features. Therefore, we treat CTGAN and KDE as two independent strategies, apply targeted augmentation to positive cases, and compare their impacts on downstream classification performance and robustness under an identical training and evaluation protocol, thereby identifying the generative approach that best matches the characteristics of our dataset.

### 4.2. Feature Selection

The dataset in this study contains 94 features, with 945 positive cases and 13,581 negative cases. Relative to the sample size, the feature dimensionality is high, which may increase training costs and amplify the risk of overfitting; therefore, feature selection was applied to reduce dimensionality. Centered on statistical significance, we adopted a filter-based feature selection approach, using SelectKBest to compute the *p*-value of each feature with respect to the label, retaining only those features with *p*-values < 0.05. This approach offers four advantages: (1) high interpretability, as each retained feature has clear statistical meaning, which facilitates clinical communication and interpretation of model decisions; (2) compatibility with different data types, as appropriate tests (e.g., ANOVA, *t*-test, chi-square) can be chosen according to feature type, allowing flexible handling of mixed continuous and categorical data; (3) low computational burden, since feature screening can be completed without training a specific model, thereby effectively reducing overall computational cost; and (4) the ability to rank feature importance, as significance levels provide an initial ordering that can serve as a basis for subsequent model optimization and variable governance.

Among these methods, principal component analysis (PCA) [21] is a feature transformation technique that linearly combines the original variables into principal components. Although it facilitates compression of continuous data and structural exploration, the principal components are relatively less interpretable. PCA is less suitable for tabular data containing many categorical variables, and it further requires additional decisions regarding the number of components to retain as well as repeated validation. In a setting that emphasizes clinical interpretability, *p*-value-based filter feature selection better meets the needs of this study while balancing performance and transparency. In summary, we adopt *p*-value < 0.05 as the primary dimensionality-reduction strategy, which is more appropriate than PCA for the data characteristics and application objectives of this work.

In this study, we performed a patient ID-based fixed split into training, validation, and test sets, and the test set was held out independently prior to any model development or feature processing. Feature selection (univariate screening with *p*-value < 0.05 via SelectKBest) was fitted exclusively on the training set, and the selected feature subset was then fixed and consistently applied to the validation and test sets for subsequent model training and prediction. Throughout the pipeline, the validation and test sets did not participate in feature selection; therefore, no information leakage arising from “peeking” at validation/test data can occur. In addition, to ensure that feature selection reflects genuine clinical relevance, SelectKBest (p<0.05) was performed only on the original training set. After feature selection, KDE-synthesized samples were added to the training set to enhance generalization. Because the feature selection procedure did not reference any synthetic samples, potential synthetic bias was effectively avoided.

### 4.3. Ensemble Learning Strategies

In this study, we adopt a stacking-based classification framework [22]. The first layer consists of gradient boosting decision trees (GBDT) [23], Light Gradient Boosting Machine (LightGBM) [24], Random Forest [25], Adaptive Boosting (AdaBoost) [26], Support Vector Machine (SVM) [27], and k-Nearest Neighbor (KNN) [28] as base models to capture complex nonlinear relationships in the data. The second layer employs logistic regression [29] as the meta-model to integrate the outputs of the first layer, using a parsimonious and stable linear decision function to reduce overfitting and enhance interpretability.

Let the probability that the *m*-th base model predicts a positive outcome (14-day readmission) for case $x_{i}$ be defined as Equation (6).

(6) $$\begin{array}{c} {\hat{p}}_{m}\left(x_{i}\right)\;\;m=1,\ldots ,M \end{array}$$

where *M* denotes the total number of base models. The predicted probabilities for the same case across all base models are then combined to form the stacked feature vector, as shown in Equation (7).

(7) $$\begin{array}{c} z_{i}={\left(\hat{p_{1}}\left(x_{i}\right),\hat{p_{2}}\left(x_{i}\right),\ldots ,\hat{p_{M}}\left(x_{i}\right)\right)}^{T} \end{array}$$

Building on this, the second-layer logistic regression models the conditional probability that case $x_{i}$ is positive as Equation (8)

(8) $$\begin{array}{c} P_{r}\left(y_{i}=1|z_{i}\right)=\sigma \left(\beta_{0}+\beta^{T}z_{i}\right)=\frac{1}{1+exp(-\beta_{0}-\sum_{m=1}^{M}\beta_{m}{\hat{p}}_{m}\left(x_{i}\right))} \end{array}$$

Here, $\beta_{0}$ is the intercept term, and $\beta ={\left(\beta_{1},\ldots ,\beta_{M}\right)}^{T}$ is the weight vector for the predicted probabilities of each base model, where $\beta_{M}$ represents the marginal effect of the *m*-th base model’s output on the final log-odds. In other words, in the M-dimensional feature space spanned by $\left({\hat{p}}_{1}\left(x_{i}\right),\ldots ,{\hat{p}}_{M}\left(x_{i}\right)\right)$, the second-layer logistic regression linearly weights and calibrates the predicted probabilities of all base models, and then uses σ(·) to map $\beta_{0}+\beta^{T}z_{i}$ to a readmission probability between 0 and 1, thereby integrating the predictive signals of multiple base models into the final ensemble prediction.

In this study, we adopted GBDT, LightGBM, Random Forest, AdaBoost, SVM, and KNN as base learners. These six classifiers are among the most widely used and representative mainstream methods in modern machine learning. The primary rationale is that the benefit of stacking arises from model diversity and complementarity. Specifically, GBDT and LightGBM, both belonging to gradient-boosted decision trees, can effectively capture complex nonlinearities and interactions among clinical variables. LightGBM further offers advantages in computational efficiency and memory usage, which facilitates stable comparisons across multiple synthetic data augmentation ratios and experimental settings. Random Forest reduces variance through randomized sampling of features and instances, improving robustness to noise and data fluctuations. AdaBoost enhances discrimination of hard-to-classify cases by reweighting misclassified samples during iterative training. SVM provides strong discriminative capability in high-dimensional feature spaces under the maximum-margin principle and can capture nonlinear decision boundaries via kernel functions. KNN supplies local decision signals based on neighborhood proximity, complementing tree-based and margin-based models that may overlook localized patterns.

For the second layer, we employed Logistic Regression as the meta-learner to integrate the outputs of base models in a parsimonious and interpretable linear form. This design enables probability weighting and calibration while controlling overall model complexity to mitigate overfitting.

For hyperparameter settings, KNN and GBDT were used with the library default configurations as baseline setups. For Random Forest, LightGBM, AdaBoost, SVM, and the meta-learner, we tuned only a small set of hyperparameters that most directly control model capacity and learning strength, thereby balancing predictive performance against the risk of overfitting. Specifically, for Random Forest, we primarily adjusted the number of trees and the feature-subsampling scheme at each split. For LightGBM, we mainly tuned the learning rate and the number of boosting iterations and enabled imbalance-aware configurations (e.g., class weighting or imbalance settings). For AdaBoost, we adjusted the ensemble size and learning rate and constrained the maximum depth of weak learners to prevent excessive complexity. For SVM, we specified the kernel function form and enabled probability estimates to support second-layer integration in stacking. For the second-layer Logistic Regression, we applied L1 regularization and incorporated class weights to improve robustness and interpretability when aggregating outputs from multiple base models.

**Figure 2:**
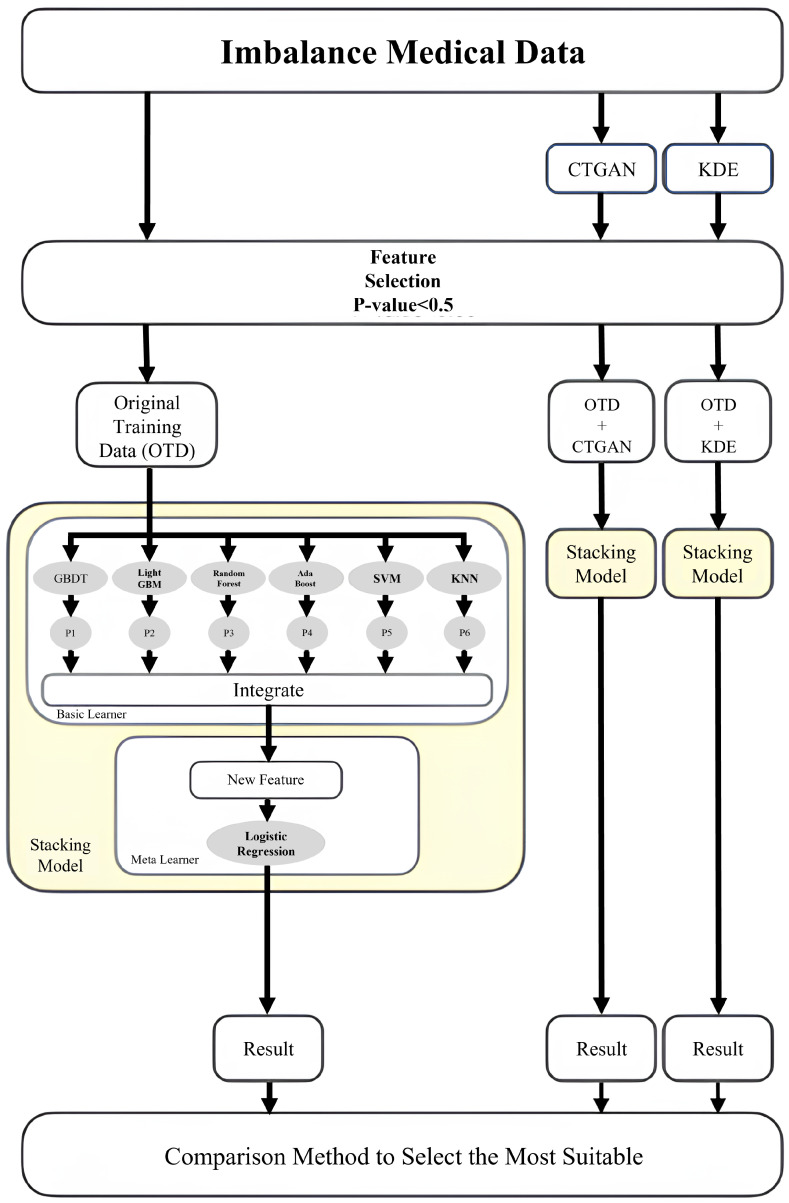
Stacking classification model.

Figure 2 illustrates the stacking workflow of this study, designed to compare the effectiveness of different data-generation strategies on imbalanced clinical data. After data preprocessing and feature selection (*p*-value < 0.05), we constructed three training scenarios: (i) the original training data (OTD), (ii) OTD combined with CTGAN-generated samples, and (iii) OTD combined with KDE-generated samples. In the first layer, base learners—including GBDT, LightGBM, Random Forest, AdaBoost, SVM, and KNN—output, for each sample, the predicted probability of “readmission within 14 days.” In the second layer, Logistic Regression aggregates these predicted probabilities by forming a stacked feature vector and produces the final readmission risk. Finally, we compared model outcomes across the three scenarios to identify the data-generation strategy and model combination that best fit our dataset.

## 5. Experimental Results

### 5.1. Data Characteristics Analysis

This study compares the distributions of clinical characteristics between patients readmitted within 14 days and those not readmitted, and evaluates the association of each variable with short-term readmission risk using odds ratios (ORs) and 95% confidence intervals Table 1. Categorical variables are presented as counts and percentages, whereas continuous variables are summarized as means and standard deviations.

Overall, the readmission and non-readmission groups showed little difference in sex distribution and most comorbidities; for example, the proportion of males was similar (81.7% vs. 77.6%; OR 1.014, 95% CI 0.841–1.222), and the confidence intervals of the ORs for most comorbidities such as diabetes mellitus, cerebrovascular disease, and chronic kidney disease all crossed 1, indicating no marked between-group differences in these characteristics. However, several clinical features were significantly associated with short-term readmission. PN (OR 1.214, 95% CI 1.059–1.392), cancer (OR 1.573, 95% CI 1.245–1.985), and the pneumococcal vaccine-related variable 2PNEU0 (OR 1.563, 95% CI 1.010–2.417) were all associated with increased readmission risk, whereas 3TRELE showed an even higher odds ratio (OR 2.520, 95% CI 1.382–4.593), possibly reflecting greater clinical complexity or severity among patients requiring this medication. By contrast, a diagnosis of hypertension was negatively associated with readmission risk (OR 0.725, 95% CI 0.579–0.907), which may be related to differences in medication management or healthcare utilization patterns.

With respect to continuous variables, age has a positive effect on readmission risk (OR 1.011, 95% CI 1.004–1.017), indicating that older patients with COPD are more likely to be readmitted within a short period. Time-related indicators of hospitalization and readmission also exhibit systematic differences: the ORs of re_ADM_TIME and avg_BED_DAY are both greater than 1, whereas that of avg_COPD_reADM_days is less than 1 (OR 0.992, 95% CI 0.992–0.993), suggesting that patients with shorter intervals between previous COPD admissions and longer average lengths of stay are at higher risk of being readmitted within 14 days. In addition, several COPD treatment and medication indicators (such as 3ALVES, 3SPI25, and the oral corticosteroid 1PREDN) also have ORs greater than 1, reflecting that patients receiving more intensive or complex treatment regimens are at higher risk of short-term readmission. Hemoglobin (HGB), by contrast, is negatively associated with readmission risk (OR 0.899, 95% CI 0.825–0.980), implying that patients with better overall hematologic and nutritional status are less likely to be readmitted in the short term.

### 5.2. Data Generation and Dimensionality Reduction

**Figure 3:**
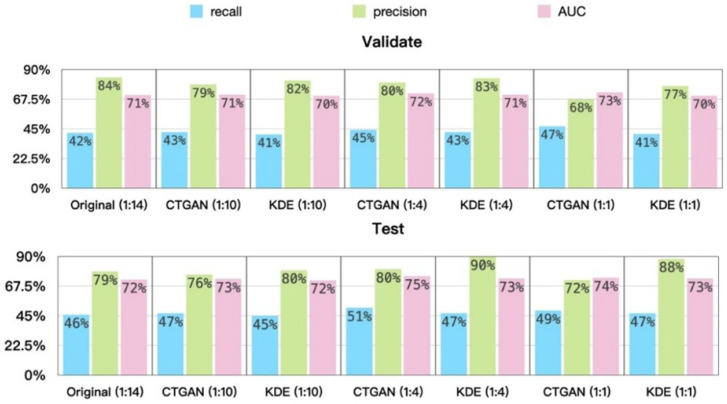
Performance of XGBoost under different generation ratios.

In terms of data augmentation, this study uses CTGAN and KDE to generate additional positive samples in the training set, adjusting the original positive-to-negative ratio from approximately 1:14 to 1:10, 1:4, and 1:1 Table 2. XGBoost (eXtreme Gradient Boosting) is then employed to evaluate performance under different generation ratios. As shown in Figure 3, differences between recall and precision can be observed: except for the test set generated using KDE, which exhibits irregular variation, all other settings show a decreasing trend as the positive–negative ratio becomes more balanced.

**Figure 4:**
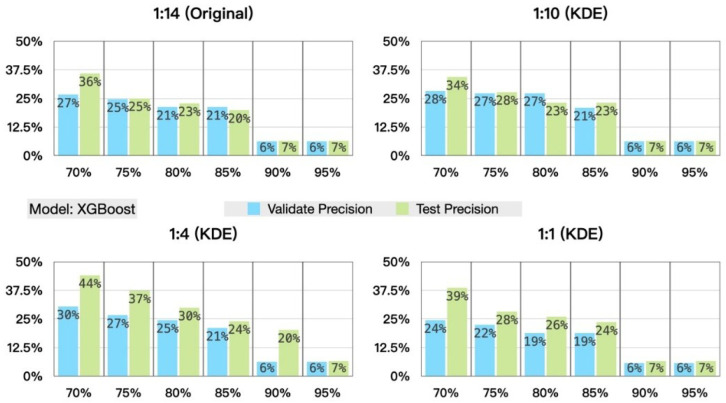
Changes in precision at fixed recall under different generation ratios in the XGBoost model.

The design objective of the model in this study is to identify at least 70% of positive cases; therefore, under the constraint that recall must be no less than 70%, we further compare the precision achieved by different methods. As shown in Figure 4, at the same recall level, the precision corresponding to a generation ratio of 1:4 is the highest. Based on these findings, subsequent experiments adopt the 1:4 generation setting as the primary focus of analysis.

At the same time, we evaluated the consistency between the distributions of generated and real data using metrics such as Maximum Mean Discrepancy (MMD) [30]. As shown in Table 3, KDE outperforms CTGAN in terms of overall distributional fit, with the generated data more closely approximating the distribution of real samples.

In addition, this study uses entropy and information gain to further assess the impact of data generation on feature signals. As shown in Table 4, after augmenting the dataset with KDE- and CTGAN-generated samples, the entropy of most features changes only slightly, but the information gain of several variables that are highly associated with readmission increases markedly. For example, for sex (SEX), the readmission time-related indicator re_ADM_TIME, and several COPD treatment medications (such as 1DL30, 1PHYLL, 1PREDN, 1PROPH, and 3ALVES), the information gain after KDE and CTGAN augmentation is higher than that in the original data. Specifically, the information gain of SEX increases from 0.24 to 0.50, that of re_ADM_TIME from 0.23 to approximately 0.45, and that of 3ALVES from 0.24 to approximately 0.48–0.50.

These features substantially overlap with the high-risk or significantly associated variables identified in the previous OR/CI analysis (such as PN, cancer, re_ADM_TIME, and several COPD medication indicators), indicating that data generation does not alter the primary risk structure present in the original data. Instead, it makes those features that are already strongly associated with 14-day readmission more readily exploitable by the model in terms of information gain from a machine learning perspective.

Building on this, we further applied *p*-value < 0.05 feature selection separately to the training set using (i) only the original data and (ii) the data augmented with generated samples, to examine whether data generation can amplify meaningful predictive signals while preserving the existing risk structure. As shown in Table 5, when only the original data are used, a total of 13 features have *p*-values < 0.05, including sex, hospitalization and readmission-related indicators, COPD medications and procedures, and clinical variables such as PN and cancer.

After incorporating KDE-generated data, the number of significant features with *p*-values < 0.05 increased to 34. Most of the original key variables—such as PN, cancer, hospitalization trajectory indicators, and COPD medications—were retained, with additional comorbidities and hematologic/biochemical indices also included.

Most of these newly added features had already exhibited trends in the OR/CI analysis that were “directionally plausible but less pronounced” or close to statistical significance. After data generation, with increases in sample size and signal strength, they were highlighted as significant variables by the *p*-value tests, indicating that KDE-generated data both preserves the known high-risk structure in the original data and amplifies weaker yet clinically plausible associations.

By comparison, after incorporating CTGAN-generated data, the number of significant features likewise increased to 35, with most of the originally significant variables retained and additional laboratory indices and environmental factors (such as ${\mathrm{NO}}_{2}$, GLU, and HDL-C) included, while overall maintaining a direction consistent with the original risk structure.

Taken together, the MMD and *p*-value-based feature selection results indicate that neither generation method disrupts the risk patterns revealed by the OR/CI analysis in the original data, whereas KDE, while preserving key features, exhibits a more pronounced effect in amplifying signals related to comorbidities and medication variables.

It can thus be inferred that data generation does not distort the underlying clinical risk structure but instead reinforces the informative signals that the model can learn. This indicates that the CTGAN/KDE-based data augmentation combined with *p*-value-based feature selection adopted in this study is indeed consistent with the findings of the preceding data characteristics analysis, providing a set of variables for the subsequent stacking ensemble model that balances clinical interpretability and predictive performance, thereby enhancing model training and improving prediction accuracy.

**Figure 5:**
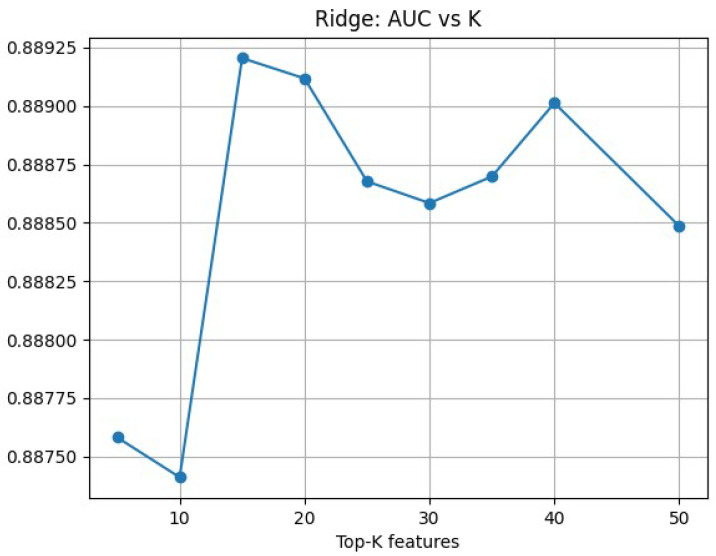
Relationship between the number of Top-K features selected by Ridge ranking and the cross-validated ROC-AUC.

Finally, we implemented LASSO and Ridge for comparison. In our experiments, LASSO retained only avg_COPD_reADM_days, which is likely attributable to the sparsity-inducing nature of L1 regularization: when features are highly correlated and predictive signals are concentrated in a small number of strong variables, the model tends to keep only the most representative predictor and shrink the remaining coefficients to zero, making it difficult to obtain a stable and sufficiently comprehensive feature set. Therefore, we further adopted Ridge with L2 regularization for feature ranking and treated *K* as a hyperparameter. Using outer cross-validation, we evaluated AUC as a function of *K* (*K* = 5–50) (Figure 5), and the results indicated that top-15 achieved the best average AUC.

Finally, under the same KDE augmentation setting and the same stacking architecture, we trained models using either the Ridge top-15 features or the 34 features selected by *p*-value + SelectKBest, and compared performance on an independent test set. The *p*-value + SelectKBest approach yielded slightly higher AUC and precision, whereas Ridge top-15 achieved slightly higher recall, as shown in Table 6. These results suggest that, under our study setting, the two feature selection strategies provide comparable discriminative performance, and the main conclusions are reasonably robust to the choice of feature selection method.

**Figure 6:**
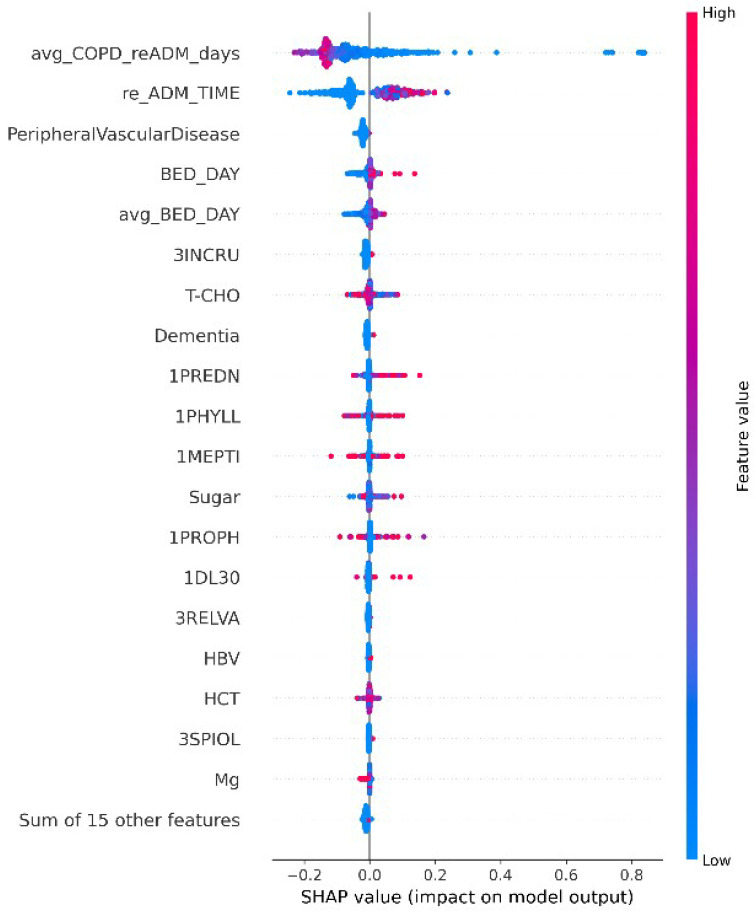
Global feature attribution of the stacking model.

In addition, we conducted a global feature-attribution analysis of the final stacking model using SHapley Additive exPlanations (SHAP) (Figure 6) to quantify, across all samples, the average magnitude and direction of each variable’s contribution to the model output. The results indicate that model decisions are primarily driven by a small set of key features. Among them, indicators related to hospitalization/readmission history—such as avg_COPD_reADM_days, re_ADM_TIME, and length-of-stay-related variables—exhibit the highest overall contributions. The distribution of feature values across the positive and negative SHAP ranges further reveals consistent trends of risk elevation or attenuation, highlighting that these care-trajectory signals play a dominant role in the model’s assessment of 14-day readmission risk.

### 5.3. Experimental Results and Analysis

**(1)**  **Prediction Results on the Validation and Test Sets**

In this study, the evaluation thresholds were set at recall levels of 70%, 75%, 80%, 85%, 90%, and 95% or higher. For each threshold, we selected the configuration with the highest F1-score to compare the performance of different models and data generation strategies. The core rationale of this strategy is that, in the task of predicting COPD readmission, it is essential to prioritize the identification of as many patients as possible who will be readmitted within 14 days (high recall), while maintaining precision (PPV) at an acceptable level. The classification models compared in this study include XGBoost (XGB), LightGBM (LGBM), AdaBoost (Ada), Random Forest (RF), and the proposed stacking classifier (SC). Their performance on the validation set was evaluated under multiple scenarios: without generated data, with CTGAN-generated data, and with KDE-generated data.

For dataset partitioning, we first randomly sampled a subset of hospitalization records from the overall cohort to construct the validation set. Cases in the validation set could partially overlap with, or be entirely distinct from, those in the training set; its primary role was model hyperparameter tuning and threshold selection. The test set, in contrast, was obtained by randomly sampling at the patient level, ensuring that patients in the test set did not appear in the training set. This design was intended to simulate the real-world scenario of predicting 14-day readmission risk for newly encountered patients and to evaluate the generalization ability of the models.

Given that, in theory, KDE is less capable than CTGAN in generating categorical variables, this study further examined the actual contribution of categorical comorbidity and vaccination variables to predictive performance. We split the features into four combinations: (1) all features, (2) excluding vaccination variables, (3) excluding comorbidities, and (4) excluding both vaccination and comorbidity variables. Under each combination, we evaluated the predictive performance of the original data, CTGAN-generated data, and KDE-generated data.

Table 7 summarizes the performance of each classification model on the validation set at the recall threshold of ≥70% across four feature configurations (all features, excluding vaccination variables, excluding comorbidities, and excluding both vaccination and comorbidity variables) and three data settings (original data, CTGAN-generated data, and KDE-generated data).

Across the four feature combinations and three data settings, the SC model combined with KDE-generated data consistently achieved relatively high PPV, F1-score, and F2-score. Among these, the SC model with KDE-generated data under the feature configuration excluding vaccination variables (the “no-vaccination” group) performed best: at a recall of 0.76, the specificity, PPV, F1, and F2 increased to 0.96, 0.55, 0.64, and 0.70, respectively. Compared with the “all features” configuration under the same KDE + SC setting, this model showed substantial improvements in positive predictive value and overall evaluation metrics, indicating that, while maintaining a high recall, it can still achieve a favorable balance between precision and overall discriminative performance. By contrast, under the same feature combination, the SC model trained on the original data and that using CTGAN-generated data both exhibited markedly lower PPV and F1-score than KDE.

Under high-recall settings, Table 8 highlights differences in the stability of the various models under a “do-not-miss” configuration. When recall is pushed to an extreme level, XGB and LGBM exhibit a sharp drop in PPV to 0.06 or values close to this level, indicating a clear “model collapse” phenomenon. In contrast, under the same threshold, the SC model can still maintain a PPV of approximately 0.20–0.34 together with a better F1-score, demonstrating that the stacking architecture is more robust under stringent recall requirements and less likely to lose discriminative ability due to threshold adjustments.

The feature combination excluding vaccination variables, when paired with KDE-generated data, achieves superior overall performance, performs well under high-recall conditions, and is less prone to model collapse. The findings indicate that minority-class samples generated by KDE can effectively strengthen the discriminative signals of each base model within the heterogeneous stacking ensemble, thereby mitigating the adverse impact of class imbalance on F1 and PPV. At the same time, they also show that removing vaccination variables does not weaken the model’s ability to capture cases of readmission within 14 days; rather, it helps reduce interference from signals less related to the target outcome, enabling the model to focus more on clinical and care-related features that are highly associated with the short-term readmission risk of COPD.

**Figure 7:**
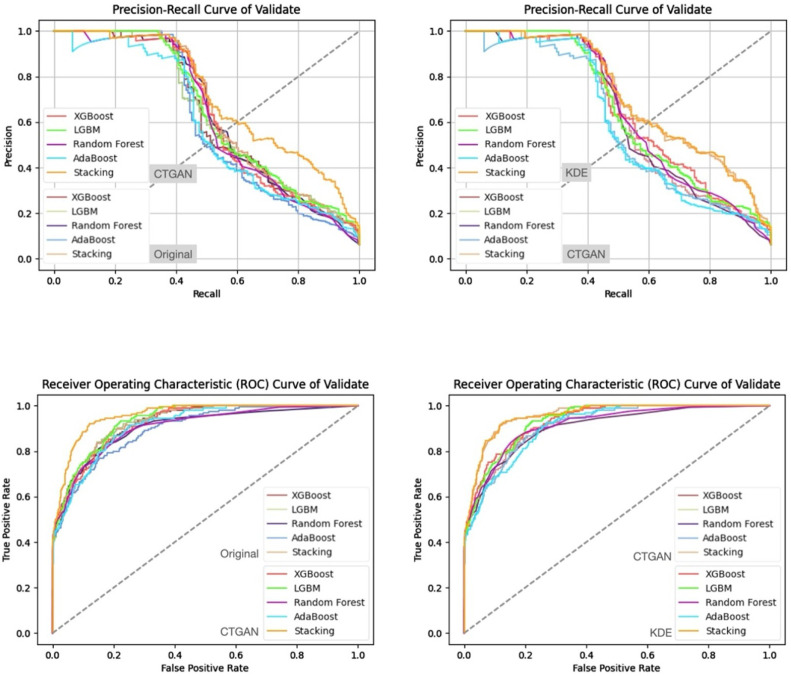
Precision–recall and ROC curves of each model on the validation set with all features.

**Figure 8:**
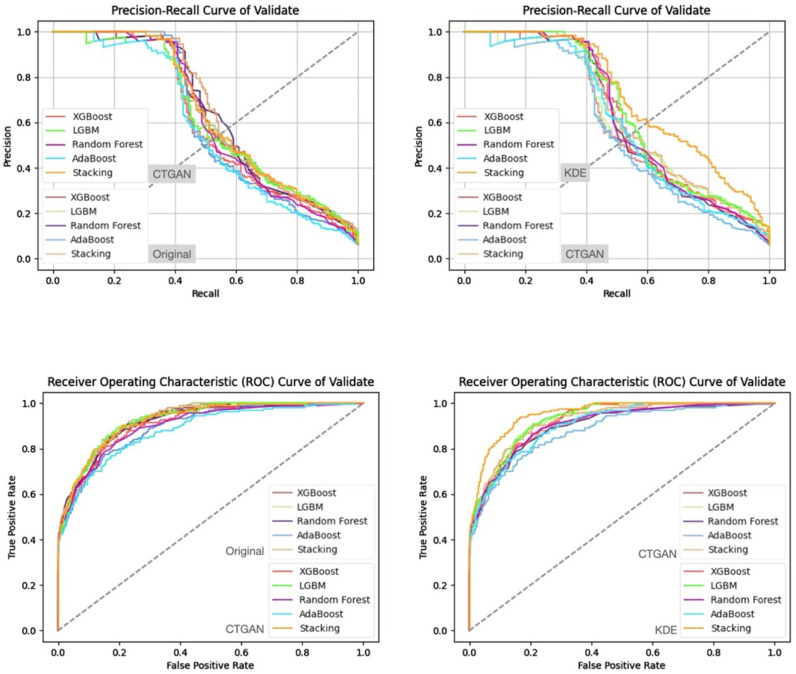
Precision–recall and ROC curves of each model on the validation set without vaccination features.

**Figure 9:**
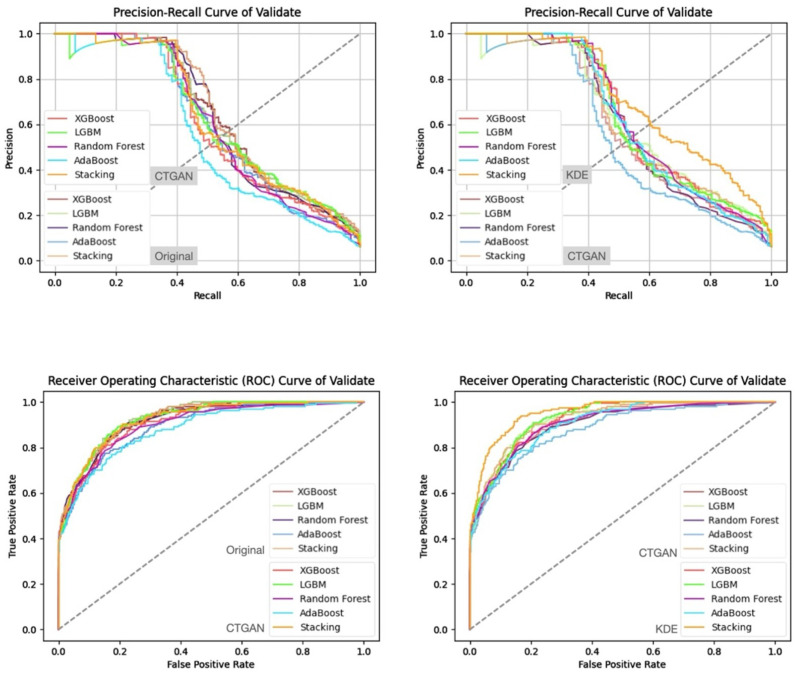
Precision–recall and ROC curves of each model on the validation set without comorbidity features.

**Figure 10:**
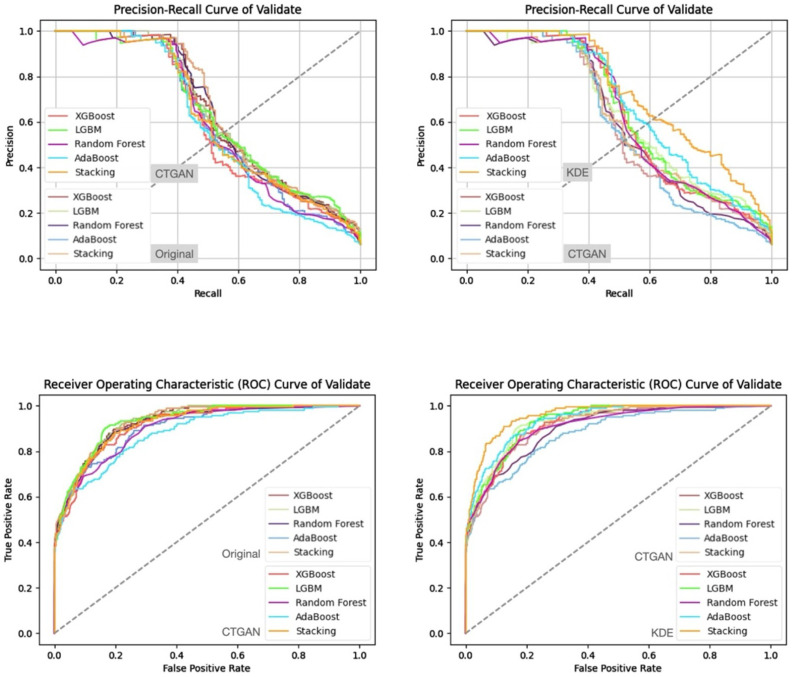
Precision–recall and ROC curves of each model on the validation set without vaccination and comorbidity features.

In addition, from the precision–recall curves and ROC curves under each feature set and data generation scenario (Figure 7, Figure 8, Figure 9 and Figure 10), it can be further observed that the PR curves of the SC model combined with KDE-generated data lie above those of the other models across most recall ranges, and its area under the ROC curve is also the largest. Taken together, the tabular and curve-based results confirm that, for this imbalanced COPD dataset, the combination of “stacking + KDE with vaccination features excluded” is the optimal configuration for simultaneously achieving high recall, high precision, and stability, which is also consistent with the earlier MMD analysis showing that KDE yields higher generation quality than CTGAN.

To validate the generalization ability of the proposed stacking model, we evaluated it on an independent test set composed entirely of patients not included in either the training or validation sets. Overall, the test-set performance metrics were similar to those on the validation set, without evident performance degradation, indicating that the stacking model maintains good stability and consistency under different data splits. This finding suggests that the stacking model developed in this study not only performs well during the training and validation phases but also preserves strong predictive performance when applied to entirely new, previously unseen test cases, further supporting its generalizability and the reliability of its practical application.

Table 9 summarizes the performance of each classification model on the test set at the recall threshold of ≥80% across four feature configurations (all features, excluding vaccination variables, excluding comorbidities, and excluding both vaccination and comorbidity variables) and three data settings (original data, CTGAN-generated data, and KDE-generated data).

**Figure 11:**
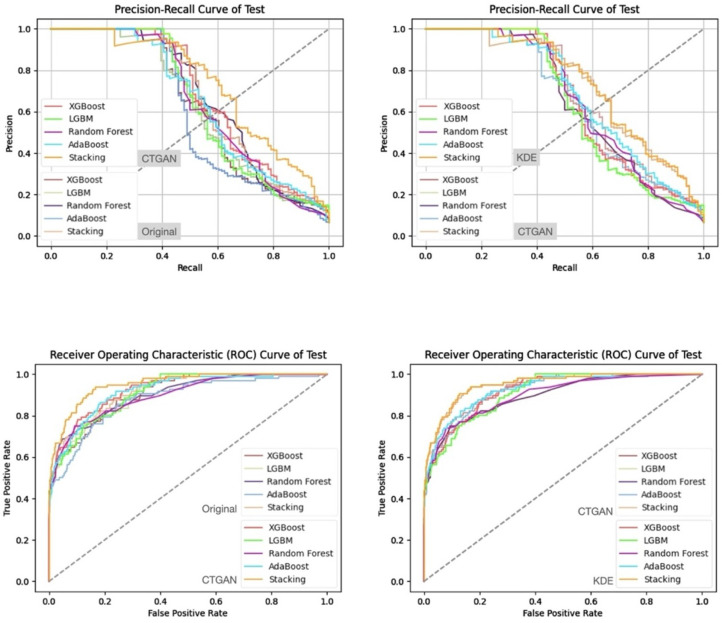
Precision–recall curves of each model on the test set with all features.

**Figure 12:**
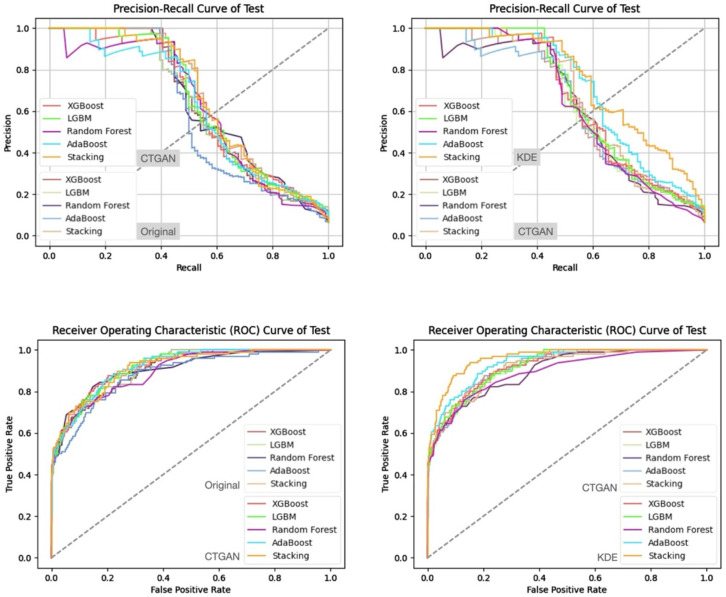
ROC curves of each model on the test set without vaccination features.

**Figure 13:**
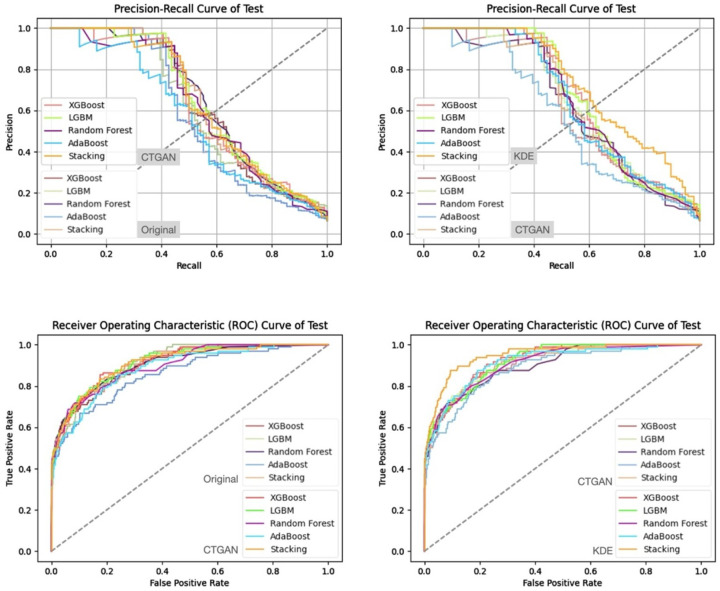
ROC curves of each model on the test set without comorbidity features.

**Figure 14:**
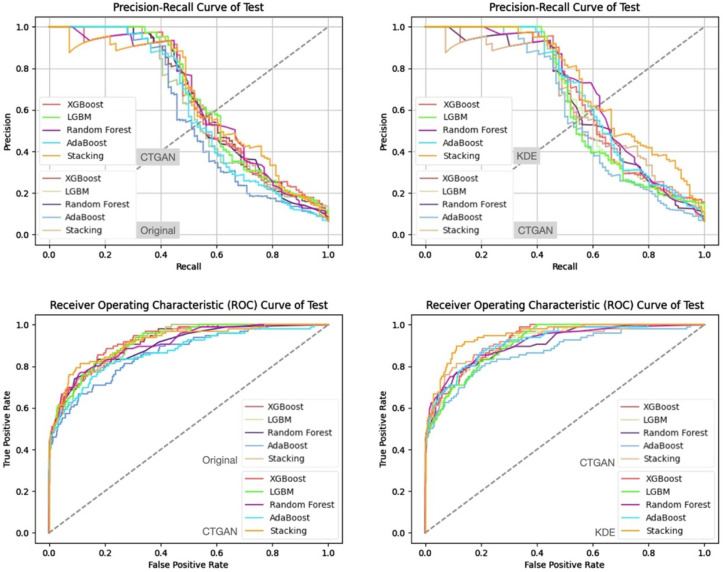
ROC curves of each model on the test set without vaccination and comorbidity features.

Compared with stricter thresholds such as recall ≥90% or 95%, this threshold avoids extreme situations in which the specificity of some baseline models approaches zero and the positive predictive value (PPV) drops sharply, and it better reflects the stable predictive capability of each model in practical applications. At recall ≥80%, the performance of stacking + KDE on the validation set across the four feature combinations is concentrated around recall ≈ 0.8, specificity ≈ 0.9, PPV ≈ 0.37–0.42, and F2-score ≈ 0.65–0.68; most metrics are superior or at least not inferior to those of the best single model in each group, indicating that, under an emphasis on high recall, specificity and precision can still be balanced. When the recall threshold is further raised to 90% or 95%, the specificity and PPV of most conventional models decline markedly, and in some combinations a “prediction collapse” phenomenon emerges, with specificity nearly dropping to zero and PPV falling to around 0.07. In contrast, although stacking combined with KDE also pays a cost in terms of specificity, it can still maintain relatively reasonable PPV and F2-score, and the precision–recall and ROC curves exhibit trends consistent with the above findings (Figure 11, Figure 12, Figure 13 and Figure 14).

**Figure 15:**
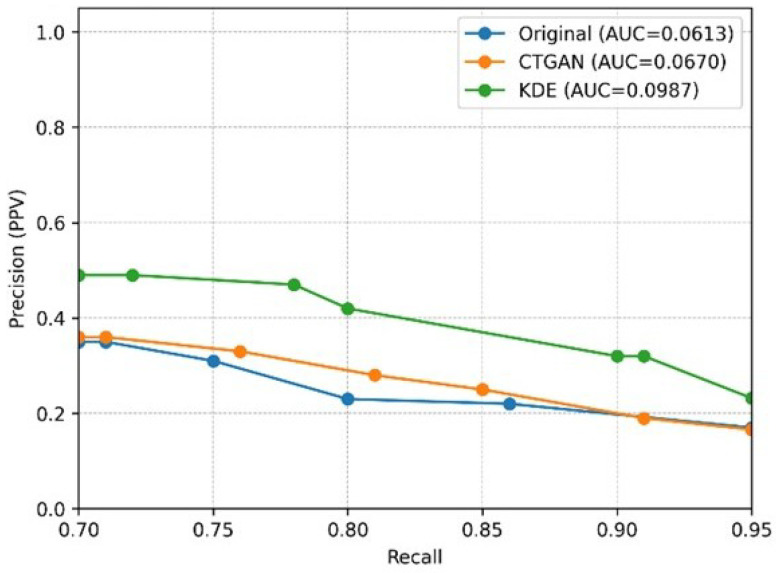
Partial PR curves (Recall 0.70–0.95) of Stacking (SC) on the test set.

Finally, because the intended clinical use case of this study requires maintaining acceptable positive predictive reliability under high-recall operation (Recall ≥ 0.70), we computed and reported the partial PR-AUC within the high-recall range (Recall 0.70–0.95) to focus on the operating region most relevant to practical decision-making. Notably, the partial PR-AUC spans only a 0.25-wide recall interval; therefore, its numerical scale is expected to be smaller than that of the full PR-AUC computed over the complete 0–1 recall range. Our results show that, on the test set for the stacking model (SC), KDE-augmented data achieve a higher partial PR-AUC (0.70–0.95) than both the Original and CTGAN settings (Figure 15), indicating better overall precision under the same high-recall requirement. This metric thus more directly reflects the practical utility of the model for clinical deployment under severe class imbalance.

In addition, we employed the DeLong test to assess the statistical significance of differences in ROC-AUC. For example, when comparing the stacking model with XGBoost, the stacking model achieved an AUC of 0.9495, whereas XGBoost achieved an AUC of 0.9178. The DeLong test indicated that this difference was statistically significant (*p* = 0.00135), supporting a significant improvement of the stacking approach over the baseline model, as shown in Table 10.

**(2)**  **Prediction Results of the Stratified Experiments**

Based on the aforementioned experimental results, this study has demonstrated that the stacking model combined with KDE-generated data achieves the best overall performance under high-recall settings. To further examine whether there is still room for optimization under this optimal configuration, we investigated the impact of stratification strategies on model performance. According to clinical relevance and population distribution, patients were divided into three groups: (1) severity levels 1–2, (2) severity levels 3–4, and (3) age ≥ 65 years. We then compared, under the Stacking + KDE setting, the predictive performance of each subgroup at different recall thresholds.

The rationale for selecting these three stratification schemes is as follows: First, cases with severity levels 3–4 account for approximately 77% of all positive cases and represent more severe conditions that are of particular clinical concern. Second, although severity levels 1–2 constitute a smaller proportion of the overall positive population, their within-stratum positive rates (7.15% for level 1 and 9.34% for level 2) are higher than that of severity level 3 (5.84%), indicating that there is a non-negligible risk of readmission even among patients with milder disease. Third, because patients with COPD are predominantly older adults, and approximately 79% of the positive samples in this study are aged 65 years or older, evaluating the predictive performance of this elderly subgroup as an independent stratum is also of practical relevance.

First, we examined the baseline effect of stratification under the stacking model using only the original data (without generated samples) (Table 11). The results show that the non-stratified group (ALL) and the severity ≥ 3 group have similar overall performance and are slightly superior to the severity ≤ 2 group in terms of AUC and F1-score; although the age ≥ 65 group achieves higher precision and F1-score, its recall is noticeably lower than that of ALL and the severity ≥ 3 group.

Table 12 summarizes the best performance of Stacking + KDE on the validation set across different recall thresholds for each subgroup under the two feature configurations “without vaccination” and “without vaccination and comorbidities.” Overall, the “without vaccination” configuration outperforms “without vaccination and comorbidities,” consistent with the non-stratified analysis. Among the stratification strategies, the severity ≥ 3 group achieves higher PPV, F1, and F2 at most thresholds than the non-stratified and other stratified groups, followed by the severity ≤ 2 group. In contrast, for the age ≥ 65 group, PPV and F1 are generally lower than those of the non-stratified setting across most recall thresholds, indicating that stratification based solely on age does not effectively enhance discriminative ability under high-recall conditions. In summary, under the Stacking + KDE framework, the “non-stratified” and “severity ≥ 3” strategies constitute more effective and stable modeling options, with the severity ≥ 3 group performing best, whereas the age ≥ 65 group is not recommended as a stand-alone stratification scheme.

To examine the generalizability of the above conclusions, we further repeated the same analysis on the test set (Table 13). The results show that, at recall ≥ 70% and higher thresholds, the PPV, F1, and F2 of the non-stratified group and the severity ≥ 3 group on the test set are very close to those on the validation set, with only limited differences, indicating that these two modeling strategies exhibit relatively stable performance on external data. In contrast, for the severity ≤ 2 and age ≥ 65 groups, PPV and F1 at the same recall thresholds decline markedly compared with the validation set, with the PPV difference exceeding 20 percentage points in some scenarios, suggesting that these two stratification schemes are more sensitive to sample variation and have relatively poorer generalizability.

Taken together, the validation and test results indicate that stratification strategies do not necessarily improve predictive performance in all scenarios. For the primary objective of this study—identifying short-term readmission cases under high-recall conditions—the non-stratified model under the “no-vaccination features + Stacking + KDE” framework already provides strong overall performance. If further improvement in PPV and F1/F2 is desired while focusing on clinically higher-risk populations, developing a dedicated model based on the severity ≥ 3 stratum offers a favorable balance of effectiveness and stability. By contrast, the severity ≤ 2 and age ≥ 65 strata are more suitable as exploratory findings that require additional data and variables for support before being adopted in practical applications.

**(2)**  **Prediction Results of the Stratified Experiments**

Based on the preceding experimental results, KDE-generated data are more suitable for this dataset than CTGAN, particularly when combined with stacking, where they yield the best performance. Although CTGAN combined with stacking outperforms most single models, its overall performance still falls short of KDE + stacking. To clarify whether this difference arises from interactions between specific base models in the stacking framework and KDE-generated data, we further conducted an ablation analysis of the stacking structure. Given that KDE emphasizes smooth estimation of local probability densities, CTGAN is more adept at preserving high-dimensional complex dependencies, and KNN is highly sensitive to local neighborhood distributions, we hypothesized that KNN may be the key component driving the advantage of “KDE + stacking.” To test this hypothesis, we used the best-performing feature configuration without vaccination variables as the basis and, under different recall thresholds, removed KNN and other base models from the stacking model in turn to systematically evaluate the marginal contribution of each base model within the stacking structure.

Table 14 and Table 15 show that, under the KDE + stacking setting, removing KNN leads to a marked decline in PPV, with the maximum difference across recall thresholds reaching approximately 21 percentage points. By contrast, in the CTGAN-generated data, removing KNN affects PPV by only about 1–6%, indicating a relatively limited marginal contribution. Notably, even after KNN is removed, the overall performance of KDE combined with stacking still exceeds that of CTGAN in most cases, suggesting that the structure of this dataset is more favorable to generation mechanisms such as KDE that emphasize local distributions.

Specifically, under extreme class imbalance, minority samples are often diluted in the feature space by the overwhelming majority class. As a result, when KNN constructs neighborhood sets, they are more likely to be dominated by majority-class instances, leading to low and unstable PPV for minority-class identification. By sampling from a locally smoothed density estimate, KDE tends to generate additional samples in the vicinity of the original minority instances, thereby improving local coverage and increasing neighborhood density for the minority class. This mechanism provides a coherent explanation for why removing KNN under KDE augmentation yields the largest drops in PPV and F1 in our ablation results. In contrast, CTGAN emphasizes learning nonlinear dependencies in high-dimensional features and the mixed-type structure of tabular fields; its generated samples are not necessarily optimized for “neighborhood-density reinforcement.” Consequently, its marginal benefit for a neighborhood-based classifier such as KNN may be less pronounced, which is consistent with the smaller ablation effects observed under CTGAN augmentation in this study.

From the ablation results of the other base models, under the KDE-generated data, removing any base model almost invariably leads to a decrease in PPV when the recall threshold is below 80%. When the recall threshold exceeds 80%, however, removing Random Forest or SVM slightly improves PPV in certain scenarios, suggesting that under extremely high recall requirements, some models may introduce noise and that appropriately pruning them can help improve precision. By contrast, in the CTGAN-generated data, the impact of removing a single model on PPV is minor and lacks a clear pattern, with noticeable gains appearing only on the test set when recall is above 80%. This indicates that CTGAN combined with stacking is relatively less sensitive to the composition of base classifiers.

To compare the contributions of stacking versus single classifiers, we further examined the standalone performance of each base model at the same recall threshold (Recall > 70%) (Table 16 and Table 17). Under both CTGAN- and KDE-generated data, SVM trained as a single model consistently exhibited very high recall but extremely low specificity and PPV, with overall F1 and F2 scores clearly inferior to those of the other models. However, when SVM was incorporated into the stacking framework—particularly in the KDE-generated data with target recall set between 70% and 80%—it instead contributed an approximate 3–13% improvement in PPV. This result indicates that the strength of stacking lies in combining complementary signals rather than relying solely on the single best model; even classifiers that perform relatively poorly on their own may still make a substantive contribution to the final prediction within an ensemble architecture.

Taken together, the above analyses indicate that, when designing a stacking architecture, it is essential to concurrently consider (1) the data generation mechanism and distributional characteristics of the data, and (2) the complementarity among base models, rather than simply selecting the “best model” based on individual performance metrics or blindly increasing the number of models. Under the clinical requirements specified in this study, when the target recall falls within the 70–80% range, the “full Stacking + KDE” configuration represents the currently optimal combination. If future clinical application scenarios demand even higher recall (e.g., ≥85% or ≥90%), the ablation results presented in this section may be used to judiciously assess the removal of models such as Random Forest or SVM, in order to achieve a more favorable balance between PPV and overall predictive performance while maintaining high recall.

Finally, to examine whether the proposed synthetic-data strategy remains advantageous under different generation paradigms, we additionally investigated an alternative approach for addressing class imbalance that emphasizes sample distances and neighborhood relationships. Specifically, we adopted A Synthetic Over-Sampling Method with Minority and Majority Classes for Imbalance Problems (SOMM) [31] as a representative distance- and neighborhood-based method and compared it with KDE. To ensure a fair comparison, KDE and SOMM were implemented and evaluated under exactly the same experimental settings, including the same patient-level data split, the same feature set, the same Stacking framework (with the same base learners and meta-learner), and the same training and evaluation pipeline. As shown in Table 18, on the test set, KDE achieved a ROC-AUC of 0.949, whereas SOMM achieved a ROC-AUC of 0.935; moreover, KDE also yielded higher Precision, Recall, and F1-score for the positive class than SOMM. Notably, although SOMM did not outperform KDE, the performance gap between the two methods was modest. Under the current dataset and experimental configuration, KDE demonstrated a slight overall advantage over SOMM. For clarity, these results are presented solely as an experimental contrast between KDE and SOMM to address the suggestion of including a non-GAN, distance-based comparison; this paragraph does not further infer whether CTGAN is a necessary condition. Meanwhile, the effectiveness of a given synthetic-data strategy may vary with the underlying data distribution and feature composition; therefore, SOMM may exhibit stronger performance under other data characteristics or application scenarios.

## 6. Conclusions

COPD is the third leading cause of death worldwide and in Taiwan, affecting more than 200 million people globally, with a continuously increasing prevalence in Taiwan. Among COPD-related outcomes, short-term readmission within 14 days is a key indicator that reflects both patient vulnerability and the quality of care. This study proposes an ensemble-learning framework integrated with data-generation techniques to address class imbalance in clinical prediction tasks. By combining a stacking ensemble with synthetic data generation, our method mitigates the classification challenge arising from the rarity of readmission events in clinical datasets, thereby enabling the development of an early warning system for predicting the risk of 14-day readmission among patients with COPD. Our results indicate that KDE-generated synthetic samples strengthen minority-class signals and, when coupled with a heterogeneous stacking ensemble, can maintain both higher precision and better stability under high-recall operating points, outperforming single classifiers and CTGAN-based augmentation. Consistently, data-quality evaluations also show that KDE-synthesized data are superior to CTGAN, and the feature sets selected from the generated data and the original data are almost identical, demonstrating that synthetic augmentation does not alter the intrinsic characteristics of the original dataset.

In the ablation study of the ensemble model, results show that KNN contributes more substantially under the KDE-augmented setting. Removing KNN in the KDE generation scenario leads to a pronounced decrease in PPV, with a maximum drop of 21%. Moreover, in some KDE experiments with recall > 80%, removing Random Forest or SVM yields a slight rebound in PPV; in contrast, under CTGAN augmentation, changes in PPV before and after removing each base learner are not markedly different. Based on these observations, we hypothesize that KDE-generated samples better preserve local density and neighborhood structure, thereby allowing neighborhood-based models (e.g., KNN) to provide more informative discrimination signals. Overall, the effectiveness of stacking is governed by model complementarity rather than the sheer number of models, and it should be tuned in accordance with recall targets. Under the recall range of 70–80% considered in this study, full stacking with KDE is the optimal configuration.

For 14-day COPD readmission prediction, most prior studies still favor a single classifier combined with conventional resampling approaches (e.g., SMOTE and its variants). In contrast, under the same clinical task and a unified evaluation protocol, this study systematically compares the feasibility and differences in two generative balancing strategies (CTGAN and KDE), providing a comparative analysis of generative imbalance-handling methods in the context of short-term readmission prediction.

Finally, under the best configuration (excluding vaccine features + stacking + KDE), we further evaluated the effect of cohort-specific modeling by stratifying patients into three clinically meaningful subgroups—severity 1–2, severity 3–4, and age ≥ 65—and comparing predictive performance across different recall thresholds. Across both the validation and test sets, the stratification strategy does not yield consistent improvements in all scenarios; however, when focusing on higher-risk cohorts, building a dedicated model for “severity ≥ 3” can further improve PPV and F1/F2 while maintaining stability, thereby providing empirical guidance on when stratification is beneficial and how to stratify.

In summary, this study integrates generative data balancing with heterogeneous ensemble learning, compares the effectiveness of different generative strategies under a consistent training and evaluation pipeline, and further provides empirical evidence for model-combination design and recall-threshold tuning through ablation and stratified analyses. Overall, the proposed framework can sustain high recall while simultaneously improving PPV, F1/F2, and generalizability, offering practical value for early warning of 14-day COPD readmission.

## Figures and Tables

**Table 1:** Descriptive statistical analysis of COPD.

Characteristic	reADM Within 14 Days (*N* = 919)	No reADM Within 14 Days (*N* = 13,607)	Odds Ratio (95% CI)
**Categorical variables, No. (%)**			
Male sex	751 (81.7%)	10563 (77.6%)	1.014 (0.841–1.222)
3INCRU	6 (0.7%)	103 (0.8%)	0.967 (0.379–2.465)
3RELV2	3 (0.3%)	40 (0.3%)	1.096 (0.311–3.862)
3SPIOL	31 (3.4%)	311 (2.3%)	1.240 (0.818–1.880)
3SR160	2 (0.2%)	56 (0.4%)	0.394 (0.095–1.641)
3TRELE	13 (1.4%)	81 (0.6%)	2.520 (1.382–4.593)
TABACCO	0 (0.0%)	2 (0.0%)	0.000 (0.000 to inf)
PN	477 (51.9%)	6393 (47.0%)	1.214 (1.059–1.392)
Hypertension	95 (10.3%)	1816 (13.3%)	0.725 (0.579–0.907)
MyocardialInfraction	13 (1.4%)	136 (1.0%)	1.403 (0.786–2.503)
CongestiveHeartFailure	77 (8.4%)	974 (7.2%)	1.243 (0.972–1.590)
PeripheralVascularDisease	1 (0.1%)	38 (0.3%)	0.391 (0.053–2.862)
CerebrovascularDiease	19 (2.1%)	340 (2.5%)	0.913 (0.570–1.462)
Dementia	4 (0.4%)	45 (0.3%)	1.419 (0.507–3.971)
DiabetesMellitus	115 (12.5%)	1885 (13.9%)	0.951 (0.776–1.166)
Hyperlipidemia	6 (0.7%)	153 (1.1%)	0.662 (0.290–1.511)
ChronicKidneyDisease	28 (3.0%)	534 (3.9%)	0.798 (0.541–1.178)
Cancer	89 (9.7%)	944 (6.9%)	1.573 (1.245–1.985)
HIV/AIDS	1 (0.1%)	11 (0.1%)	1.150 (0.147–9.003)
AlcoholLiverDisease	24 (2.6%)	386 (2.8%)	0.899 (0.588–1.374)
TB	19 (2.1%)	193 (1.4%)	1.439 (0.893–2.319)
HBV	15 (1.6%)	339 (2.5%)	0.668 (0.395–1.130)
HCV	18 (2.0%)	203 (1.5%)	1.393 (0.852–2.278)
2PNEU0	23 (2.5%)	216 (1.6%)	1.563 (1.010–2.417)
2PNEUM	9 (1.0%)	74 (0.5%)	1.634 (0.806–3.312)
2PRE13	35 (3.8%)	354 (2.6%)	1.408 (0.982–2.018)
2PRE30	0 (0.0%)	0 (0.0%)	-
2SYNFL	0 (0.0%)	0 (0.0%)	-
**COPD Level**			
1	158.0 (17.2%)	2120.0 (15.6%)	1.125 (0.071–1.343)
2	57.0 (6.2%)	564.0 (4.1%)	1.529 (0.071–2.025)
3	615.0 (66.9%)	10191.0 (74.9%)	0.678 (0.100–0.782)
4	89.0 (9.7%)	732.0 (5.4%)	1.886 (0.069–2.376)
**Continuous variables, mean (SD)**			
CO	0.44 (0.14%)	0.44 (0.14%)	0.042 (0.010–0.181)
O_3_	29.31 (7.31%)	29.14 (7.66%)	1.011 (0.998–1.024)
SO_2_	4.29 (1.57%)	4.27 (1.58%)	0.922 (0.869–0.978)
NO	3.68 (1.81%)	3.62 (1.79%)	1.011 (0.860–1.188)
NO_2_	16.90 (6.11%)	16.52 (6.22%)	1.081 (0.945–1.236)
NOx	20.58 (7.57%)	20.16 (7.61%)	1.006 (0.877–1.154)
PM2.5	29.04 (12.74%)	28.87 (13.37%)	1.009 (0.995–1.023)
PM10	59.46 (21.67%)	59.33 (23.31%)	0.994 (0.986–1.002)
AGE	73.01 (11.00%)	72.84 (12.00%)	1.011 (1.004–1.017)
re_ADM_TIME	2.96 (4.01%)	1.53 (2.74%)	1.032 (1.014–1.050)
BED_DAY	11.80 (10.09%)	10.08 (10.40%)	1.008 (0.999–1.018)
3SPI25	0.15 (0.36%)	0.11 (0.32%)	1.278 (1.043–1.566)
avg_BED_DAY	11.12 (7.92%)	9.80 (8.29%)	1.015 (1.001–1.029)
1DL30	1.25 (8.44%)	1.10 (7.83%)	1.001 (0.993–1.009)
1MEPTI	8.75 (19.11%)	7.26 (17.47%)	1.001 (0.997–1.004)
1PHYLL	14.19 (23.33%)	10.51 (20.75%)	1.003 (1.000–1.007)
1PREDN	10.90 (26.28%)	6.73 (18.35%)	1.007 (1.004–1.010)
1PROPH	9.22 (23.78%)	10.98 (25.05%)	0.997 (0.994–1.000)
3ALVES	0.04 (0.20%)	0.02 (0.14%)	1.793 (1.213–2.651)
3ANORO	0.06 (0.23%)	0.04 (0.20%)	1.253 (0.909–1.727)
3RELVA	0.02 (0.13%)	0.02 (0.15%)	0.796 (0.463–1.368)
avg_COPD_reADM_days	97.41 (158.24%)	851.34 (875.23%)	0.992 (0.992–0.993)
3VENTO	0.13 (0.34%)	0.12 (0.32%)	0.865 (0.700–1.068)
%sO2c	94.03 (6.33%)	94.08 (6.36%)	0.999 (0.988–1.010)
ALB	7.11 (81.66%)	4.63 (24.32%)	1.001 (1.000–1.002)
BNP	103.97 (82.32%)	110.86 (180.54%)	1.000 (0.999–1.000)
BUN	17.42 (14.25)	17.41 (13.85)	1.001 (0.995–1.006)
CEA	2.38 (4.64)	6.64 (466.76)	0.998 (0.991–1.005)
CK	119.58 (73.66)	120.38 (266.90)	1.000 (1.000–1.000)
CK-MB	8.96 (5.09)	8.92 (7.77)	1.002 (0.994–1.010)
CRP	9.99 (27.21)	10.47 (28.33)	0.998 (0.995–1.001)
CRTN	1.03 (0.29)	1.04 (0.40)	0.888 (0.718–1.097)
Ca++	9.22 (1.26)	9.23 (1.23)	0.991 (0.940–1.046)
Cortisol	10.47 (3.95)	10.55 (4.87)	0.996 (0.980–1.012)
D-Dimer	0.28 (0.30)	0.33 (2.43)	0.949 (0.849–1.061)
GLU	88.75 (23.98)	87.91 (24.64)	1.002 (0.999–1.005)
GPT	24.33 (15.33)	24.59 (37.90)	1.000 (0.997–1.002)
HCT	39.66 (6.28)	39.89 (6.17)	1.028 (0.996–1.060)
HDL-C	90.27 (23.84)	88.51 (24.15)	1.003 (1.000–1.006)
HGB	12.90 (2.05)	13.02 (2.01)	0.899 (0.825–0.980)
HbA1C	3.03 (1.70)	3.04 (1.79)	1.001 (0.963–1.039)
IgE	56.59 (61.60)	58.65 (77.82)	0.999 (0.998–1.001)
K	4.02 (0.56)	4.06 (0.57)	0.882 (0.777–1.000)
LDL-C	76.23 (14.95)	75.95 (16.39)	1.002 (0.998–1.006)
Lactate	1.37 (0.50)	1.38 (0.78)	1.020 (0.910–1.142)
Mg	2.01 (0.30)	2.00 (0.30)	1.040 (0.797–1.356)
Mite DF	25.44 (14.22)	24.76 (14.37)	1.003 (0.999–1.008)
Mite/far	24.96 (14.54)	24.92 (14.49)	1.000 (0.996–1.005)
O2SAT	97.14 (3.49)	97.27 (2.74)	0.985 (0.964–1.006)
SCC	1.07 (1.11)	1.02 (0.70)	1.069 (0.993–1.150)
Sugar	90.54 (27.64)	93.39 (62.78)	0.998 (0.996–1.000)
T-BIL	0.71 (0.70)	0.68 (0.63)	1.039 (0.966–1.118)
T-CHO	149.15 (29.10)	150.42 (29.38)	0.999 (0.994–1.003)
T3	139.48 (34.18)	140.13 (34.97)	1.000 (0.000 to inf)
T4	139.48 (34.18)	140.13 (34.97)	1.000 (0.000 to inf)
TCO2	26.60 (3.84)	26.59 (4.69)	0.997 (0.981–1.013)
TG	79.76 (41.14)	81.22 (43.01)	0.999 (0.998–1.001)
TP	7.34 (0.56)	7.35 (0.56)	0.981 (0.868–1.108)
TSH	2.27 (1.13)	2.25 (2.84)	1.002 (0.979–1.024)
Theophy	0.67 (1.39)	0.61 (1.15)	1.032 (0.985–1.081)
Tropo-I	0.02 (0.02)	0.06 (1.24)	0.209 (0.023–1.867)
WBC	8.98 (3.46)	8.74 (4.30)	1.012 (0.999–1.026)
hsCRP	0.51 (0.29)	0.50 (0.29)	1.073 (0.863–1.334)
pCO2	41.75 (7.90)	41.51 (8.58)	1.003 (0.994–1.011)
House_dust	0.00 (0.07)	0.00 (0.10)	1.005 (0.436–2.318)

**Table 2:** Generation ratios for training data.

Training Data and Generated Sample Ratios	1:14 (Original)	1:10	1:4	1:1
Real positive data/percentage	659 (100%)	659 (67.4%)	659 (26.9%)	659 (6.7%)
Generated positive data/percentage	0 (0.0%)	319 (32.6%)	1787 (73.1%)	9126 (93.3%)
Total positive cases	659	978	2446	9785
Total negative cases	9785	9785	9785	9785
Total training data	10,444	10,763	12,231	19,570
Proportion of generated data	0.0%	3.0%	14.6%	46.6%

**Table 3:** MMD values for generated data.

Metric	CTGAN	KDE
MMD	0.00218	0.00117

**Table 4:** Entropy (E) and Information Gain (IG) Before and After Adding Synthetic Data.

Feature	Original (E)	KDE (E)	CTGAN (E)	Original (IG)	KDE (IG)	CTGAN (IG)
CO	1.88	2.01	2.00	0.07	0.06	0.06
$O_{3}$	1.81	1.85	1.85	0.07	0.06	0.06
${\mathrm{SO}}_{2}$	1.57	1.69	1.62	0.07	0.06	0.06
NO	1.58	1.62	1.58	0.07	0.06	0.06
${\mathrm{NO}}_{2}$	1.99	2.04	2.04	0.07	0.06	0.06
NOx	1.92	2.01	2.01	0.07	0.06	0.06
PM2.5	1.95	1.96	1.96	0.07	0.06	0.06
PM10	1.97	2.03	2.03	0.07	0.06	0.06
AGE	1.78	1.78	1.78	0.23	0.20	0.20
SEX	0.53	0.54	0.53	0.24	0.50	0.50
re_ADM_TIME	0.59	0.52	0.52	0.23	0.45	0.43
BED_DAY	0.14	0.13	0.13	0.23	0.26	0.26
avg_COPD_reADM_days	1.83	1.68	1.68	0.05	0.04	0.04
avg_BED_DAY	0.19	0.16	0.16	0.21	0.21	0.21
1DL30	0.15	0.13	0.13	0.24	0.46	0.49
1MEPTI	0.53	0.48	0.48	0.23	0.45	0.45
1PHYLL	0.67	0.60	0.60	0.23	0.43	0.40
1PREDN	0.32	0.28	0.28	0.23	0.41	0.46
1PROPH	0.70	0.62	0.62	0.23	0.47	0.47
3ALVES	0.10	0.18	0.12	0.24	0.48	0.50
3ANORO	0.17	0.23	0.18	0.24	0.49	0.50
3INCRU	0.04	0.14	0.05	0.24	0.47	0.50
3RELV2	0.02	0.12	0.02	0.24	0.47	0.50
3RELVA	0.10	0.18	0.10	0.24	0.48	0.50
3SPI25	0.36	0.40	0.40	0.24	0.49	0.49
3SPIOL	0.11	0.19	0.11	0.24	0.48	0.50
3SR160	0.03	0.13	0.03	0.24	0.47	0.50
3TRELE	0.04	0.13	0.04	0.24	0.47	0.50
3VENTO	0.37	0.40	0.38	0.24	0.50	0.50
copd_level	0.81	0.93	0.91	0.23	0.36	0.36
%sO2c	0.45	0.67	0.67	0.23	0.19	0.19
ALB	0.00	0.00	0.00	0.23	0.20	0.20
BNP	0.07	0.06	0.06	0.22	0.19	0.19
BUN	0.33	0.30	0.30	0.22	0.19	0.19
CEA	0.00	0.00	0.00	0.23	0.41	0.41
CK	0.00	0.00	0.00	0.23	0.19	0.19
CK-MB	0.00	0.00	0.00	0.23	0.30	0.30
CRP	0.26	0.23	0.23	0.19	0.26	0.26
CRTN	0.12	0.17	0.17	0.23	0.36	0.38
Ca++	1.12	1.28	1.28	0.23	0.20	0.20
Cortisol	0.01	0.01	0.01	0.23	0.20	0.20
D-Dimer	0.01	0.01	0.01	0.23	0.30	0.33
GLU	0.02	0.03	0.03	0.23	0.20	0.20
GPT	0.01	0.01	0.01	0.23	0.20	0.20
HCT	1.28	1.58	1.58	0.22	0.19	0.19
HDL-C	2.03	2.07	2.07	0.23	0.20	0.20
HGB	1.42	1.47	1.47	0.23	0.20	0.20
HbA1C	1.33	1.28	1.28	0.23	0.24	0.24
IgE	0.03	0.02	0.02	0.20	0.17	0.17
K	0.76	1.09	1.09	0.23	0.20	0.20
LDL-C	1.06	1.23	1.23	0.22	0.18	0.18
Lactate	0.02	0.02	0.02	0.23	0.22	0.22
Mg	1.15	1.32	1.48	0.23	0.20	0.20
Mite DF	2.30	2.23	2.23	0.22	0.19	0.19
Mite/far	2.30	2.23	2.23	0.22	0.19	0.19
O2SAT	0.06	0.45	0.45	0.23	0.20	0.20
SCC	0.03	0.03	0.03	0.23	0.31	0.31
Sugar	0.01	0.01	0.01	0.23	0.20	0.20
T-BIL	0.02	0.01	0.01	0.23	0.22	0.22
T-CHO	1.46	1.55	1.55	0.23	0.20	0.20
T3	1.37	1.54	1.54	0.07	0.06	0.06
T4	1.37	1.54	1.54	0.07	0.06	0.06
TCO2	0.18	0.57	0.57	0.23	0.19	0.19
TG	0.65	0.65	0.65	0.23	0.20	0.20
TP	1.54	1.35	1.35	0.23	0.19	0.19
TSH	0.01	0.01	0.01	0.14	0.12	0.12
Theophy	0.08	0.07	0.07	0.23	0.23	0.23
Tropo-I	0.01	0.01	0.01	0.23	0.23	0.23
WBC	0.12	0.10	0.10	0.19	0.16	0.16
hsCRP	1.30	1.35	1.36	0.08	0.07	0.07
pCO2	0.66	0.69	0.69	0.22	0.19	0.19
House_dust	0.01	0.12	0.01	0.24	0.46	0.50
COPD	0.00	0.00	0.00	0.24	0.46	0.50
TABACCO	0.00	0.11	0.00	0.24	0.47	0.50
Hypertension	0.39	0.42	0.39	0.24	0.50	0.50
PeripheralVascularDisease	0.02	0.12	0.02	0.24	0.47	0.50
PN	0.69	0.69	0.69	0.24	0.50	0.50
MyocardialInfraction	0.06	0.15	0.06	0.24	0.47	0.50
CongestiveHeartFailure	0.26	0.32	0.26	0.24	0.49	0.50
CerebrovascularDiease	0.12	0.19	0.11	0.24	0.48	0.50
Dementia	0.02	0.12	0.03	0.24	0.47	0.50
DiabetesMellitus	0.40	0.43	0.40	0.24	0.50	0.50
Hyperlipidemia	0.06	0.15	0.06	0.24	0.48	0.50
ChronicKidneyDisease	0.16	0.22	0.16	0.24	0.49	0.50
Cancer	0.26	0.32	0.26	0.24	0.49	0.50
HIV/AIDS	0.01	0.12	0.01	0.24	0.46	0.50
AlcoholLiverDisease	0.13	0.19	0.13	0.24	0.49	0.50
TB	0.08	0.16	0.08	0.24	0.48	0.50
HBV	0.11	0.18	0.11	0.24	0.49	0.50
HCV	0.08	0.17	0.08	0.24	0.48	0.50

**Table 5:** Feature selection results with *p*-value < 0.05 before and after adding generated data.

Feature Category	Selected Features (*p*-Value < 0.05)
**Original**	
Basic information	’SEX’, ’BED_DAY’, ’avg_BED_DAY’, ’avg_COPD_reADM_days’, ’re_ADM_TIME’
Drug	’1PHYLL’, ’1PREDN’, ’3ALVES’, ’3ANORO’, ’3SPI25’, ’3TRELE’
Comorbidity	’PN’, ’Cancer’
**KDE**	
Basic information	’SEX’, ’BED_DAY’, ’avg_BED_DAY’, ’avg_COPD_reADM_days’, ’re_ADM_TIME’
Drug	’1DL30’, ’1MEPTI’, ’1PHYLL’, ’1PREDN’, ’1PROPH’, ’3ALVES’, ’3ANORO’, ’3INCRU’, ’3RELVA’, ’3SPI25’, ’3SPIOL’, ’copd_level’
Blood	’HCT’, ’HGB’, ’K’, ’Mg’, ’Sugar’, ’T-CHO’
Comorbidity	’PN’, ’Hypertension’, ’CongestiveHeartFailure’, ’PeripheralVascularDisease’, ’CerebrovascularDiease’, ’Dementia’, ’DiabetesMellitus’, ’Hyperlipidemia’, ’ChronicKidneyDisease’, ’HBV’, ’Cancer’
**CTGAN**	
Air quality	’No2’
Basic information	’SEX’, ’BED_DAY’, ’avg_BED_DAY’, ’avg_COPD_reADM_days’, ’re_ADM_TIME’
Drug	’1DL30’, ’1MEPTI’, ’1PHYLL’, ’1PREDN’, ’1PROPH’, ’3ALVES’, ’3ANORO’, ’3RELV2’, ’3SPI25’, ’3TRELE’, ’3VENTO’, ’copd_level’
Blood	’CRTN’, ’GLU’, ’HCT’, ’HDL-C’, ’HGB’, ’SCC’, ’WBC’, ’hsCRP’, ’House_dust’
Comorbidity	’PN’, ’MyocardialInfraction’, ’CongestiveHeartFailure’, ’Dementia’, ’ChronicKidneyDisease’, ’Cancer’, ’HIV/AIDS’, ’HCV’

**Table 6:** Test-set performance comparison of feature selection methods (KDE + Stacking).

Feature Set	Split	Accuracy	Precision	Recall	F1-Score	ROC-AUC
KDE	Test	0.94	0.54	0.71	0.61	0.948894
Ridge	Test	0.93	0.48	0.72	0.58	0.947984

**Table 7:** Performance comparison of each model on the validation set at 70% recall under different feature combinations and data generation settings.

Validate	Recall	Specificity	PPV	NPV	F1 Score	F2 Score
**Original—All features**						
XGB	0.71	0.91	0.35	0.98	0.47	0.59
LGBM	0.71	0.91	0.35	0.98	0.47	0.59
Ada	0.71	0.88	0.28	0.98	0.40	0.54
RF	0.72	0.89	0.30	0.98	0.43	0.57
SC	0.71	0.91	0.35	0.98	0.47	0.59
**Original—Without vaccination**						
XGB	0.74	0.86	0.27	0.98	0.39	0.55
LGBM	0.71	0.91	0.35	0.98	0.47	0.59
Ada	0.71	0.88	0.28	0.98	0.40	0.54
RF	0.70	0.90	0.31	0.98	0.43	0.56
SC	0.71	0.92	0.38	0.98	0.49	0.60
**Original—Without comorbidities**						
XGB	0.74	0.89	0.31	0.98	0.43	0.58
LGBM	0.71	0.92	0.36	0.98	0.48	0.59
Ada	0.71	0.90	0.33	0.98	0.45	0.57
RF	0.71	0.90	0.33	0.98	0.45	0.58
SC	0.71	0.92	0.37	0.98	0.49	0.60
**Original—Without vaccination and comorbidities**						
XGB	0.71	0.91	0.36	0.98	0.48	0.60
LGBM	0.71	0.92	0.36	0.98	0.48	0.59
Ada	0.72	0.89	0.31	0.98	0.43	0.57
RF	0.72	0.89	0.30	0.98	0.43	0.56
SC	0.70	0.92	0.36	0.98	0.48	0.59
**CTGAN-generated data—All features**						
XGB	0.72	0.90	0.32	0.98	0.45	0.58
LGBM	0.70	0.91	0.34	0.98	0.46	0.58
Ada	0.73	0.83	0.22	0.98	0.34	0.50
RF	0.71	0.89	0.30	0.98	0.42	0.56
SC	0.70	0.91	0.35	0.98	0.47	0.58
**CTGAN-generated data—Without vaccination**						
XGB	0.71	0.90	0.33	0.98	0.45	0.58
LGBM	0.71	0.90	0.33	0.98	0.45	0.58
Ada	0.71	0.87	0.28	0.98	0.40	0.54
RF	0.71	0.88	0.27	0.98	0.40	0.54
SC	0.70	0.92	0.37	0.98	0.48	0.59
**CTGAN-generated data—Without comorbidities**						
XGB	0.78	0.86	0.28	0.98	0.41	0.57
LGBM	0.71	0.92	0.38	0.98	0.50	0.61
Ada	0.73	0.81	0.20	0.98	0.32	0.48
RF	0.71	0.89	0.30	0.98	0.42	0.55
SC	0.73	0.91	0.34	0.98	0.47	0.59
**CTGAN-generated data—Without vaccination and comorbidities**						
XGB	0.72	0.91	0.34	0.98	0.46	0.59
LGBM	0.73	0.90	0.33	0.98	0.45	0.58
Ada	0.71	0.90	0.32	0.98	0.44	0.57
RF	0.71	0.89	0.31	0.98	0.43	0.56
SC	0.70	0.91	0.35	0.98	0.47	0.59
**KDE-generated data—All features**						
XGB	0.70	0.90	0.32	0.98	0.44	0.57
LGBM	0.74	0.90	0.34	0.98	0.47	0.60
Ada	0.71	0.90	0.31	0.98	0.44	0.57
RF	0.70	0.92	0.37	0.98	0.48	0.59
SC	0.76	0.95	0.49	0.98	0.60	0.68
**KDE-generated data—Without vaccination**						
XGB	0.71	0.89	0.30	0.98	0.43	0.56
LGBM	0.71	0.90	0.33	0.98	0.45	0.57
Ada	0.71	0.88	0.29	0.98	0.41	0.55
RF	0.71	0.90	0.33	0.98	0.45	0.58
SC	0.76	0.96	0.55	0.98	0.64	0.70
**KDE-generated data—Without comorbidities**						
XGB	0.73	0.90	0.32	0.98	0.44	0.58
LGBM	0.72	0.91	0.36	0.98	0.48	0.60
Ada	0.72	0.90	0.34	0.98	0.46	0.59
RF	0.70	0.92	0.36	0.98	0.48	0.59
SC	0.74	0.95	0.50	0.98	0.60	0.67
**KDE-generated data—Without vaccination and comorbidities**						
XGB	0.71	0.88	0.28	0.98	0.40	0.55
LGBM	0.73	0.90	0.33	0.98	0.46	0.59
Ada	0.73	0.87	0.27	0.98	0.40	0.54
RF	0.71	0.90	0.33	0.98	0.45	0.58
SC	0.72	0.95	0.51	0.98	0.60	0.67

**Table 8:** Best performance of each model on the validation set at recall ≥ 90% (all features).

Validate	Recall	Specificity	PPV	NPV	F1 Score	F2 Score
**Original**						
XGB	1.00	0.00	0.06	NaN	0.12	0.25
LGBM	0.90	0.78	0.21	0.99	0.35	0.55
Ada	0.91	0.67	0.16	0.99	0.27	0.46
RF	0.92	0.78	0.22	0.99	0.35	0.56
SC	0.90	0.75	0.20	0.99	0.32	0.53
**CTGAN-generated data**						
XGB	0.92	0.70	0.17	0.99	0.29	0.49
LGBM	0.91	0.78	0.22	0.99	0.35	0.55
Ada	0.90	0.56	0.12	0.99	0.21	0.39
RF	0.92	0.67	0.16	0.99	0.27	0.47
SC	0.91	0.71	0.18	0.99	0.29	0.50
**KDE-generated data**						
XGB	1.00	0.00	0.06	NaN	0.12	0.25
LGBM	0.94	0.77	0.21	0.99	0.35	0.56
Ada	0.90	0.78	0.22	0.99	0.35	0.55
RF	0.93	0.65	0.15	0.99	0.26	0.46
SC	0.90	0.88	0.34	0.99	0.49	0.68

**Table 9:** Performance comparison of each model on the test set at 80% recall under different feature combinations and data generation settings.

Test	Recall	Specificity	PPV	NPV	F1 Score	F2 Score
**Original—All features**						
XGB	0.81	0.83	0.25	0.98	0.38	0.56
LGBM	0.81	0.83	0.25	0.98	0.38	0.56
Ada	0.80	0.80	0.22	0.98	0.34	0.52
RF	0.80	0.83	0.25	0.98	0.38	0.55
SC	0.80	0.81	0.23	0.98	0.35	0.53
**Original—Without vaccination**						
XGB	0.81	0.81	0.23	0.98	0.36	0.54
LGBM	0.81	0.83	0.25	0.98	0.38	0.56
Ada	0.80	0.80	0.22	0.98	0.34	0.52
RF	0.81	0.84	0.27	0.98	0.40	0.58
SC	0.81	0.82	0.24	0.98	0.38	0.55
**Original—Without comorbidities**						
XGB	0.84	0.75	0.19	0.99	0.32	0.50
LGBM	0.81	0.81	0.23	0.98	0.36	0.54
Ada	0.80	0.73	0.17	0.98	0.29	0.47
RF	0.83	0.73	0.18	0.98	0.29	0.48
SC	0.80	0.79	0.21	0.98	0.34	0.52
**Original—Without vaccination and comorbidities**						
XGB	0.83	0.75	0.19	0.98	0.31	0.50
LGBM	0.81	0.81	0.23	0.98	0.36	0.54
Ada	0.80	0.73	0.17	0.98	0.29	0.47
RF	0.80	0.86	0.29	0.98	0.42	0.59
SC	0.80	0.81	0.23	0.98	0.36	0.54
**CTGAN-generated data—All features**						
XGB	0.80	0.85	0.28	0.98	0.41	0.58
LGBM	0.81	0.84	0.26	0.98	0.40	0.57
Ada	0.80	0.79	0.21	0.98	0.34	0.52
RF	0.81	0.75	0.19	0.98	0.30	0.48
SC	0.81	0.85	0.28	0.98	0.41	0.58
**CTGAN-generated data—Without vaccination**						
XGB	0.83	0.76	0.19	0.98	0.31	0.50
LGBM	0.81	0.83	0.25	0.98	0.38	0.56
Ada	0.82	0.72	0.17	0.98	0.28	0.47
RF	0.80	0.82	0.24	0.98	0.36	0.54
SC	0.80	0.85	0.27	0.98	0.40	0.58
**CTGAN-generated data—Without comorbidities**						
XGB	0.80	0.84	0.26	0.98	0.40	0.57
LGBM	0.82	0.82	0.24	0.99	0.38	0.56
Ada	0.83	0.76	0.19	0.98	0.31	0.50
RF	0.81	0.79	0.21	0.98	0.33	0.52
SC	0.80	0.84	0.26	0.98	0.39	0.57
**CTGAN-generated data—Without vaccination and comorbidities**						
XGB	0.80	0.82	0.24	0.98	0.37	0.55
LGBM	0.80	0.86	0.28	0.98	0.42	0.59
Ada	0.81	0.79	0.21	0.98	0.34	0.52
RF	0.80	0.85	0.28	0.98	0.41	0.58
SC	0.80	0.88	0.32	0.98	0.45	0.61
**KDE-generated data—All features**						
XGB	0.84	0.81	0.24	0.99	0.37	0.56
LGBM	0.80	0.81	0.22	0.98	0.35	0.53
Ada	0.81	0.87	0.30	0.99	0.44	0.61
RF	0.82	0.80	0.22	0.98	0.35	0.54
SC	0.80	0.92	0.42	0.99	0.55	0.68
**KDE-generated data—Without vaccination**						
XGB	0.80	0.87	0.30	0.98	0.44	0.60
LGBM	0.82	0.81	0.23	0.98	0.36	0.55
Ada	0.82	0.86	0.29	0.99	0.43	0.61
RF	0.80	0.82	0.24	0.98	0.37	0.55
SC	0.81	0.91	0.38	0.99	0.51	0.66
**KDE-generated data—Without comorbidities**						
XGB	0.80	0.81	0.23	0.98	0.36	0.53
LGBM	0.81	0.82	0.24	0.98	0.37	0.55
Ada	0.80	0.84	0.26	0.98	0.39	0.57
RF	0.80	0.83	0.25	0.98	0.38	0.56
SC	0.80	0.91	0.39	0.99	0.53	0.66
**KDE-generated data—Without vaccination and comorbidities**						
XGB	0.83	0.83	0.26	0.99	0.40	0.58
LGBM	0.83	0.81	0.24	0.99	0.37	0.55
Ada	0.80	0.83	0.25	0.98	0.39	0.56
RF	0.80	0.84	0.26	0.98	0.39	0.56
SC	0.80	0.90	0.37	0.98	0.50	0.65

**Table 10:** ROC-AUC significance comparison using the DeLong test (Stacking vs. XGBoost).

Comparison	AUC (Stacking)	AUC (XGBoost)	DeLong *p*-Value
Stacking (AUC) vs. XGBoost	0.9495	0.9178	0.00135

**Table 11:** Effect of stratification on the stacking model using original data.

Group	Precision	Recall	F1-Score	AUC
ALL	0.20	0.91	0.33	0.833
Severity ≤ 2	0.16	0.84	0.27	0.746
Severity ≥ 3	0.21	0.92	0.34	0.838
Age ≥ 65	0.27	0.84	0.41	0.839

**Table 12:** Best performance of each subgroup on the validation set under Stacking + KDE at recall ≥ 70% (without vaccination/without vaccination and comorbidities).

Validate	Recall	Specificity	PPV	NPV	F1 Score	F2 Score
**Recall ≥ 70**	No vaccine/No vaccine & comorbs					
ALL	0.76/0.72	0.96/0.95	0.55/0.51	0.98/0.98	0.64/0.60	0.70/0.67
Age ≥ 65	0.75/0.71	0.96/0.94	0.53/0.42	0.98/0.98	0.63/0.52	0.70/0.62
Severity ≥ 3	0.74/0.72	0.98/0.98	0.71/0.70	0.98/0.98	0.72/0.71	0.73/0.72
Severity ≤ 2	0.80/0.78	0.94/0.94	0.53/0.52	0.98/0.98	0.64/0.62	0.73/0.71
**Recall ≥ 75**	No vaccine/No vaccine & comorbs					
ALL	0.76/0.76	0.96/0.94	0.55/0.48	0.98/0.98	0.64/0.59	0.70/0.68
Age ≥ 65	0.75/0.76	0.96/0.92	0.53/0.36	0.98/0.98	0.63/0.49	0.70/0.63
Severity ≥ 3	0.75/0.75	0.98/0.97	0.69/0.63	0.98/0.98	0.72/0.68	0.74/0.72
Severity ≤ 2	0.80/0.78	0.94/0.94	0.53/0.52	0.98/0.98	0.64/0.62	0.73/0.71
**Recall ≥ 80**	No vaccine/No vaccine & comorbs					
ALL	0.80/0.82	0.94/0.93	0.47/0.44	0.99/0.99	0.59/0.57	0.70/0.70
Age ≥ 65	0.81/0.81	0.91/0.91	0.36/0.34	0.99/0.99	0.49/0.48	0.64/0.64
Severity ≥ 3	0.87/0.81	0.96/0.95	0.59/0.52	0.99/0.99	0.71/0.64	0.80/0.73
Severity ≤ 2	0.80/0.80	0.94/0.93	0.53/0.49	0.98/0.98	0.64/0.61	0.73/0.71
**Recall ≥ 85**	No vaccine/No vaccine & comorbs					
ALL	0.85/0.86	0.92/0.91	0.40/0.38	0.99/0.99	0.55/0.53	0.70/0.69
Age ≥ 65	0.86/0.87	0.89/0.86	0.32/0.27	0.99/0.99	0.47/0.41	0.64/0.60
Severity ≥ 3	0.87/0.86	0.96/0.94	0.59/0.47	0.99/0.99	0.71/0.61	0.80/0.73
Severity ≤ 2	0.85/0.85	0.92/0.91	0.47/0.44	0.99/0.99	0.61/0.58	0.74/0.72
**Recall ≥ 90**	No vaccine/No vaccine & comorbs					
ALL	0.90/0.90	0.87/0.84	0.31/0.27	0.99/0.99	0.46/0.42	0.65/0.62
Age ≥ 65	0.91/0.90	0.83/0.80	0.24/0.21	0.99/0.99	0.38/0.35	0.58/0.55
Severity ≥ 3	0.91/0.91	0.94/0.92	0.48/0.43	0.99/0.99	0.63/0.58	0.77/0.74
Severity ≤ 2	0.90/0.90	0.90/0.86	0.44/0.36	0.99/0.99	0.59/0.51	0.74/0.69
**Recall ≥ 95**	No vaccine/No vaccine & comorbs					
ALL	0.95/0.96	0.80/0.75	0.24/0.21	1.00/1.00	0.38/0.34	0.60/0.55
Age ≥ 65	0.96/0.96	0.76/0.74	0.19/0.18	1.00/1.00	0.32/0.30	0.53/0.51
Severity ≥ 3	0.96/0.96	0.90/0.71	0.38/0.17	1.00/1.00	0.54/0.29	0.73/0.50
Severity ≤ 2	0.95/0.95	0.71/0.73	0.22/0.23	0.99/0.99	0.36/0.37	0.57/0.58

**Table 13:** Best performance of each subgroup on the test set under Stacking + KDE at recall ≥ 70% (without vaccination/without vaccination and comorbidities).

Test	Recall	Specificity	PPV	NPV	F1 Score	F2 Score
**Recall ≥ 70**	No vaccine/No vaccine & comorbs					
ALL	0.73/0.71	0.97/0.97	0.64/0.60	0.98/0.98	0.68/0.65	0.71/0.68
Age ≥ 65	0.71/0.71	0.93/0.90	0.37/0.30	0.98/0.98	0.49/0.42	0.60/0.56
Severity ≥ 3	0.71/0.71	0.97/0.97	0.66/0.63	0.98/0.98	0.68/0.67	0.70/0.69
Severity ≤ 2	0.70/0.70	0.88/0.88	0.40/0.38	0.96/0.96	0.51/0.49	0.61/0.60
**Recall ≥ 75**	No vaccine/No vaccine & comorbs					
ALL	0.75/0.76	0.97/0.95	0.61/0.52	0.98/0.98	0.67/0.62	0.72/0.70
Age ≥ 65	0.75/0.75	0.89/0.88	0.30/0.28	0.98/0.98	0.43/0.40	0.58/0.56
Severity ≥ 3	0.79/0.78	0.95/0.95	0.55/0.54	0.98/0.98	0.65/0.64	0.73/0.72
Severity ≤ 2	0.77/0.77	0.78/0.84	0.27/0.35	0.97/0.97	0.40/0.48	0.56/0.62
**Recall ≥ 80**	No vaccine/No vaccine & comorbs					
ALL	0.81/0.80	0.91/0.90	0.38/0.37	0.99/0.98	0.51/0.50	0.66/0.65
Age ≥ 65	0.80/0.80	0.83/0.85	0.23/0.25	0.99/0.99	0.36/0.38	0.54/0.55
Severity ≥ 3	0.81/0.86	0.95/0.93	0.54/0.47	0.99/0.99	0.64/0.60	0.73/0.73
Severity ≤ 2	0.80/0.87	0.74/0.80	0.25/0.32	0.97/0.98	0.38/0.47	0.56/0.65
**Recall ≥ 85**	No vaccine/No vaccine & comorbs					
ALL	0.85/0.85	0.89/0.87	0.35/0.31	0.99/0.99	0.50/0.46	0.66/0.63
Age ≥ 65	0.86/0.86	0.80/0.80	0.21/0.22	0.99/0.99	0.34/0.35	0.54/0.54
Severity ≥ 3	0.88/0.86	0.93/0.93	0.46/0.47	0.99/0.99	0.60/0.60	0.74/0.73
Severity ≤ 2	0.90/0.87	0.56/0.80	0.18/0.32	0.98/0.98	0.31/0.47	0.51/0.65
**Recall ≥ 90**	No vaccine/No vaccine & comorbs					
ALL	0.92/0.91	0.80/0.83	0.24/0.28	0.99/0.99	0.39/0.42	0.59/0.62
Age ≥ 65	0.91/0.91	0.74/0.74	0.18/0.18	0.99/0.99	0.30/0.30	0.50/0.50
Severity ≥ 3	0.93/0.91	0.89/0.90	0.37/0.40	0.99/0.99	0.53/0.55	0.72/0.72
Severity ≤ 2	0.90/0.90	0.56/0.69	0.18/0.25	0.98/0.98	0.31/0.39	0.51/0.59
**Recall ≥ 95**	No vaccine/No vaccine & comorbs					
ALL	0.96/0.96	0.76/0.73	0.22/0.20	1.00/1.00	0.35/0.33	0.57/0.54
Age ≥ 65	0.95/0.95	0.69/0.60	0.16/0.13	1.00/1.00	0.28/0.23	0.48/0.42
Severity ≥ 3	0.96/0.96	0.82/0.78	0.28/0.24	1.00/1.00	0.43/0.38	0.64/0.60
Severity ≤ 2	1.00/1.00	0.19/0.25	0.12/0.13	1.00/1.00	0.22/0.23	0.41/0.42

**Table 14:** Comparison of the impact of removing each model from stacking on the validation set at recall ≥ 70%.

Validate	Recall	Specificity	PPV	NPV	F1 Score	F2 Score
**Recall ≥ 70**	**CTGAN/** **KDE**					
Stacking	0.70/0.76	0.92/0.96	0.37/0.55	0.98/0.98	0.48/0.64	0.59/0.70
n_Ada	0.70/0.70	0.91/0.95	0.35/0.50	0.98/0.98	0.46/0.59	0.58/0.65
n_GBDT	0.73/0.74	0.90/0.95	0.34/0.49	0.98/0.98	0.46/0.59	0.59/0.67
n_KNN	0.73/0.73	0.91/0.91	0.36/0.36	0.98/0.98	0.48/0.48	0.60/0.60
n_LGBM	0.71/0.70	0.90/0.95	0.32/0.50	0.98/0.98	0.44/0.58	0.57/0.65
n_RF	0.75/0.76	0.90/0.94	0.34/0.48	0.98/0.98	0.46/0.59	0.60/0.68
n_SVM	0.76/0.76	0.90/0.94	0.35/0.46	0.98/0.98	0.48/0.58	0.61/0.67
**Recall ≥ 75**	**CTGAN/** **KDE**					
Stacking	0.79/0.76	0.88/0.96	0.30/0.55	0.98/0.98	0.44/0.64	0.60/0.70
n_Ada	0.76/0.78	0.88/0.93	0.30/0.43	0.98/0.98	0.43/0.55	0.58/0.67
n_GBDT	0.76/0.76	0.90/0.94	0.33/0.48	0.98/0.98	0.46/0.59	0.60/0.68
n_KNN	0.77/0.76	0.90/0.90	0.33/0.34	0.98/0.98	0.46/0.47	0.61/0.61
n_LGBM	0.76/0.83	0.87/0.92	0.27/0.42	0.98/0.99	0.40/0.56	0.56/0.70
n_RF	0.75/0.76	0.90/0.94	0.34/0.48	0.98/0.98	0.46/0.59	0.60/0.68
n_SVM	0.76/0.76	0.90/0.94	0.35/0.46	0.98/0.98	0.48/0.58	0.61/0.67
**Recall ≥ 80**	**CTGAN/** **KDE**					
Stacking	0.84/0.80	0.86/0.94	0.28/0.47	0.99/0.99	0.42/0.59	0.60/0.70
n_Ada	0.80/0.82	0.87/0.92	0.29/0.41	0.99/0.99	0.43/0.55	0.59/0.69
n_GBDT	0.80/0.80	0.85/0.93	0.26/0.45	0.98/0.99	0.39/0.58	0.57/0.69
n_KNN	0.80/0.82	0.87/0.87	0.29/0.30	0.99/0.99	0.43/0.44	0.59/0.61
n_LGBM	0.85/0.83	0.82/0.92	0.24/0.42	0.99/0.99	0.38/0.56	0.57/0.70
n_RF	0.82/0.80	0.87/0.93	0.30/0.43	0.99/0.99	0.44/0.56	0.61/0.69
n_SVM	0.81/0.81	0.87/0.93	0.29/0.44	0.99/0.99	0.42/0.57	0.59/0.69
**Recall ≥ 85**	**CTGAN/** **KDE**					
Stacking	0.85/0.85	0.83/0.92	0.25/0.40	0.99/0.99	0.39/0.55	0.57/0.70
n_Ada	0.85/0.85	0.83/0.89	0.25/0.34	0.99/0.99	0.38/0.49	0.57/0.66
n_GBDT	0.85/0.85	0.82/0.91	0.24/0.39	0.99/0.99	0.37/0.53	0.56/0.69
n_KNN	0.86/0.85	0.81/0.84	0.23/0.26	0.99/0.99	0.37/0.40	0.56/0.59
n_LGBM	0.85/0.85	0.82/0.91	0.24/0.38	0.99/0.99	0.38/0.53	0.57/0.68
n_RF	0.87/0.85	0.84/0.91	0.26/0.40	0.99/0.99	0.40/0.54	0.59/0.69
n_SVM	0.85/0.85	0.84/0.91	0.27/0.38	0.99/0.99	0.41/0.52	0.59/0.68
**Recall ≥ 90**	**CTGAN/** **KDE**					
Stacking	0.91/0.90	0.76/0.87	0.20/0.31	0.99/0.99	0.33/0.46	0.53/0.65
n_Ada	0.91/0.91	0.77/0.85	0.21/0.29	0.99/0.99	0.34/0.44	0.54/0.64
n_GBDT	0.91/0.91	0.74/0.87	0.19/0.31	0.99/0.99	0.32/0.47	0.52/0.66
n_KNN	0.91/0.90	0.76/0.76	0.21/0.20	0.99/0.99	0.33/0.33	0.54/0.53
n_LGBM	0.90/0.91	0.73/0.87	0.19/0.32	0.99/0.99	0.31/0.48	0.51/0.67
n_RF	0.90/0.91	0.75/0.88	0.19/0.34	0.99/0.99	0.32/0.49	0.52/0.68
n_SVM	0.91/0.91	0.76/0.88	0.20/0.33	0.99/0.99	0.33/0.48	0.54/0.67
**Recall ≥ 95**	**CTGAN/** **KDE**					
Stacking	0.95/0.95	0.60/0.80	0.14/0.24	0.99/1.00	0.24/0.38	0.44/0.60
n_Ada	0.98/0.95	0.52/0.82	0.12/0.26	1.00/1.00	0.21/0.41	0.40/0.62
n_GBDT	0.95/0.95	0.60/0.83	0.14/0.27	0.99/1.00	0.24/0.42	0.44/0.63
n_KNN	0.96/0.96	0.66/0.65	0.16/0.16	1.00/1.00	0.27/0.27	0.47/0.47
n_LGBM	0.95/0.95	0.63/0.78	0.15/0.23	0.99/1.00	0.25/0.37	0.45/0.58
n_RF	0.95/0.95	0.62/0.83	0.15/0.28	0.99/1.00	0.25/0.43	0.45/0.64
n_SVM	0.95/0.96	0.62/0.82	0.14/0.27	0.99/1.00	0.25/0.42	0.45/0.63

**Table 15:** Comparison of the impact of removing each model from stacking on the test set at recall ≥ 70%.

Test	Recall	Specificity	PPV	NPV	F1 Score	F2 Score
**Recall ≥ 70**	**CTGAN/** **KDE**					
Stacking	0.71/0.73	0.91/0.97	0.36/0.64	0.98/0.98	0.48/0.68	0.59/0.71
n_Ada	0.71/0.71	0.91/0.93	0.35/0.43	0.98/0.98	0.47/0.53	0.59/0.63
n_GBDT	0.71/0.71	0.92/0.94	0.39/0.47	0.98/0.98	0.50/0.56	0.61/0.64
n_KNN	0.71/0.71	0.92/0.94	0.39/0.47	0.98/0.98	0.50/0.56	0.61/0.64
n_LGBM	0.71/0.73	0.91/0.94	0.37/0.48	0.98/0.98	0.48/0.58	0.60/0.66
n_RF	0.71/0.72	0.92/0.96	0.38/0.53	0.98/0.98	0.49/0.61	0.60/0.67
n_SVM	0.71/0.72	0.92/0.95	0.37/0.52	0.98/0.98	0.49/0.60	0.60/0.67
**Recall ≥ 75**	**CTGAN/** **KDE**					
Stacking	0.75/0.75	0.88/0.97	0.31/0.61	0.98/0.98	0.43/0.67	0.58/0.72
n_Ada	0.75/0.81	0.89/0.90	0.33/0.37	0.98/0.99	0.46/0.51	0.60/0.66
n_GBDT	0.75/0.79	0.90/0.93	0.33/0.42	0.98/0.98	0.46/0.55	0.60/0.67
n_KNN	0.75/0.76	0.91/0.92	0.36/0.41	0.98/0.98	0.48/0.53	0.62/0.65
n_LGBM	0.75/0.75	0.89/0.93	0.33/0.43	0.98/0.98	0.45/0.55	0.60/0.65
n_RF	0.78/0.75	0.85/0.94	0.27/0.48	0.98/0.98	0.40/0.59	0.57/0.67
n_SVM	0.75/0.75	0.91/0.94	0.36/0.48	0.98/0.98	0.49/0.58	0.62/0.67
**Recall ≥ 80**	**CTGAN/** **KDE**					
Stacking	0.80/0.81	0.85/0.91	0.27/0.38	0.98/0.99	0.40/0.51	0.58/0.66
n_Ada	0.80/0.81	0.84/0.90	0.25/0.37	0.98/0.99	0.39/0.51	0.56/0.66
n_GBDT	0.80/0.82	0.83/0.92	0.25/0.41	0.98/0.99	0.38/0.55	0.56/0.69
n_KNN	0.80/0.81	0.84/0.88	0.26/0.32	0.98/0.99	0.39/0.46	0.56/0.62
n_LGBM	0.80/0.80	0.84/0.91	0.26/0.39	0.98/0.99	0.39/0.52	0.57/0.66
n_RF	0.81/0.81	0.83/0.92	0.25/0.41	0.98/0.99	0.39/0.54	0.56/0.68
n_SVM	0.80/0.81	0.83/0.92	0.24/0.42	0.98/0.99	0.37/0.55	0.55/0.68
**Recall ≥ 85**	**CTGAN/** **KDE**					
Stacking	0.85/0.85	0.75/0.89	0.19/0.35	0.99/0.99	0.31/0.50	0.50/0.66
n_Ada	0.85/0.85	0.76/0.87	0.20/0.32	0.99/0.99	0.32/0.46	0.52/0.64
n_GBDT	0.85/0.86	0.75/0.89	0.19/0.35	0.99/0.99	0.31/0.50	0.51/0.67
n_KNN	0.91/0.85	0.72/0.84	0.19/0.27	0.99/0.99	0.31/0.42	0.51/0.60
n_LGBM	0.85/0.86	0.76/0.89	0.20/0.36	0.99/0.99	0.32/0.51	0.51/0.67
n_RF	0.86/0.86	0.79/0.90	0.22/0.38	0.99/0.99	0.36/0.53	0.55/0.69
n_SVM	0.86/0.86	0.76/0.90	0.20/0.37	0.99/0.99	0.33/0.52	0.52/0.68
**Recall ≥ 90**	**CTGAN/** **KDE**					
Stacking	0.91/0.92	0.71/0.80	0.18/0.24	0.99/0.99	0.30/0.39	0.50/0.59
n_Ada	0.92/0.91	0.64/0.85	0.15/0.29	0.99/0.99	0.26/0.44	0.46/0.64
n_GBDT	0.91/0.91	0.70/0.87	0.17/0.32	0.99/0.99	0.29/0.47	0.49/0.66
n_KNN	0.91/0.92	0.72/0.79	0.19/0.23	0.99/0.99	0.31/0.37	0.51/0.58
n_LGBM	0.91/0.91	0.71/0.85	0.18/0.30	0.99/0.99	0.30/0.45	0.50/0.65
n_RF	0.91/0.92	0.72/0.86	0.19/0.31	0.99/0.99	0.31/0.47	0.51/0.66
n_SVM	0.91/0.91	0.72/0.86	0.18/0.31	0.99/0.99	0.31/0.46	0.51/0.65
**Recall ≥ 95**	**CTGAN/** **KDE**					
Stacking	0.97/0.96	0.61/0.76	0.15/0.22	1.00/1.00	0.26/0.35	0.46/0.57
n_Ada	0.97/0.96	0.50/0.75	0.12/0.21	1.00/1.00	0.21/0.34	0.40/0.56
n_GBDT	0.96/0.96	0.62/0.72	0.15/0.19	1.00/1.00	0.26/0.32	0.46/0.53
n_KNN	0.98/0.96	0.63/0.72	0.16/0.19	1.00/1.00	0.27/0.32	0.48/0.53
n_LGBM	0.96/0.97	0.64/0.69	0.16/0.18	1.00/1.00	0.27/0.30	0.47/0.51
n_RF	0.96/0.96	0.62/0.80	0.15/0.25	1.00/1.00	0.26/0.40	0.46/0.61
n_SVM	0.96/0.97	0.63/0.77	0.15/0.23	1.00/1.00	0.26/0.37	0.47/0.58

**Table 16:** Training results of single models on the validation set.

Valid	Recall	Specificity	PPV	NPV	F1 Score	F2 Score
**Base Model**	**CTGAN/** **KDE**					
n_Ada	0.71/0.71	0.87/0.88	0.28/0.29	0.98/0.98	0.40/0.41	0.54/0.55
n_GBDT	0.73/0.72	0.91/0.91	0.34/0.34	0.98/0.98	0.47/0.47	0.59/0.59
n_KNN	0.76/0.70	0.78/0.92	0.19/0.38	0.98/0.98	0.30/0.49	0.47/0.60
n_LGBM	0.71/0.71	0.90/0.90	0.33/0.33	0.98/0.98	0.45/0.45	0.58/0.57
n_RF	0.71/0.71	0.88/0.90	0.27/0.33	0.98/0.98	0.40/0.45	0.54/0.58
SVM	1.00/0.98	0.00/0.39	0.06/0.10	-/1.00	0.12/0.18	0.25/0.35

**Table 17:** Training results of single models on the test set.

Test	Recall	Specificity	PPV	NPV	F1 Score	F2 Score
**Base Model**	**CTGAN/** **KDE**					
n_Ada	0.73/0.72	0.83/0.92	0.23/0.39	0.98/0.98	0.35/0.51	0.51/0.62
n_GBDT	0.72/0.72	0.90/0.90	0.34/0.34	0.98/0.98	0.46/0.46	0.59/0.59
n_KNN	0.72/0.88	0.77/0.85	0.18/0.28	0.98/0.99	0.29/0.43	0.45/0.62
n_LGBM	0.73/0.72	0.91/0.90	0.35/0.34	0.98/0.98	0.47/0.46	0.60/0.59
n_RF	0.72/0.71	0.88/0.92	0.30/0.39	0.98/0.98	0.42/0.50	0.56/0.61
n_SVM	1.00/0.98	0.00/0.38	0.07/0.10	-/1.00	0.12/0.18	0.26/0.35

**Table 18:** Comparison of 14-day readmission prediction performance between SOMM and KDE on the test set.

Method	ROC-AUC	Precision	Recall	F1	Specificity	Accuracy
SOMM	0.9346	0.35	0.77	0.48	0.899	0.89
KDE	0.9491	0.42	0.80	0.55	0.923	0.92

## Data Availability

The data used in this study were obtained from internal records of Kaohsiung Medical University Hospital. Due to the inclusion of personal and sensitive patient information, the data are protected under Taiwan’s Personal Data Protection Act and related regulations, and therefore cannot be made publicly available without the consent of the data subjects. Reasonable requests for access to the data should be directed to Da-Wei Wu (Email: u8900030@gmail.com) and will be considered subject to institutional approval and applicable ethical and legal requirements.
